# Primaquine dose and the risk of haemolysis in patients with uncomplicated *Plasmodium vivax* malaria: a systematic review and individual patient data meta-analysis

**DOI:** 10.1016/S1473-3099(23)00431-0

**Published:** 2024-02

**Authors:** Megha Rajasekhar, Julie A Simpson, Benedikt Ley, Peta Edler, Cindy S Chu, Tesfay Abreha, Ghulam R Awab, J Kevin Baird, Germana Bancone, Bridget E Barber, Matthew J Grigg, Jimee Hwang, Harin Karunajeewa, Marcus V G Lacerda, Simone Ladeia-Andrade, Alejandro Llanos-Cuentas, Sasithon Pukrittayakamee, Komal R Rijal, Kavitha Saravu, Inge Sutanto, Walter R J Taylor, Kamala Thriemer, James A Watson, Philippe J Guerin, Nicholas J White, Ric N Price, Robert J Commons, WorldWide Antimalarial Resistance Network (WWARN) Vivax Primaquine Dosing Efficacy, Tolerability and Safety Study Group, Bipin Adhikari, Mohammad Shafiul Alam, Nicholas M Anstey, Ashenafi Assefa, Sarah C Boyd, Nguyen Hoang Chau, Nicholas PJ Day, Tamiru Shibiru Degaga, Arjen M Dondorp, Marcelo Urbano Ferreira, Prakash Ghimire, Justin A Green, Wasif Ali Khan, Gavin CKW Koh, Asrat Hailu Mekuria, Mohammad Nader Naadim, Erni J Nelwan, Francois Nosten, Ayodhia Pitaloka Pasaribu, David J Price, Kasia Stepniewska, Lorenz von Seidlein, Timothy William, Charles J Woodrow, Adugna Woyessa

**Affiliations:** a Centre for Epidemiology and Biostatistics, Melbourne School of Population and Global Health, The University of Melbourne, Melbourne, VIC, Australia; b WorldWide Antimalarial Resistance Network (WWARN), Asia-Pacific Regional Centre, Melbourne, VIC, Australia; c Global Health Division, Menzies School of Health Research and Charles Darwin University, Darwin, NT, Australia; d Department of Infectious Diseases, The University of Melbourne at the Peter Doherty Institute for Infection and Immunity, Melbourne, VIC, Australia; e Shoklo Malaria Research Unit, Mahidol Oxford Tropical Medicine Research Unit (MORU), Faculty of Tropical Medicine, Mahidol University, Mae Sot, Thailand; f Centre for Tropical Medicine and Global Health, Nuffield Department of Medicine, University of Oxford, Oxford, UK; g ICAP, Columbia University Mailman School of Public Health, Addis Ababa, Ethiopia; h MORU, Faculty of Tropical Medicine, Mahidol University, Bangkok, Thailand; i Department of Clinical Tropical Medicine, Faculty of Tropical Medicine, Mahidol University, Bangkok, Thailand; j Nangarhar Medical Faculty, Nangarhar University, Jalalabad, Afghanistan; k Oxford University Clinical Research Unit Indonesia, Faculty of Medicine Universitas Indonesia, Jakarta, Indonesia; l QIMR Berghofer Medical Research Institute, Brisbane, QLD, Australia; m Infectious Diseases Society Sabah-Menzies School of Health Research Clinical Research Unit, Kota Kinabalu, Malaysia; n US President's Malaria Initiative, Malaria Branch, US Centers for Disease Control and Prevention, Atlanta, GA, USA; o Institute for Global Health Sciences, University of California San Francisco, San Francisco, CA, USA; p Department of Medicine-Western Health, Melbourne Medical School, The University of Melbourne, St Albans, VIC, Australia; q Fundação de Medicina Tropical Dr Heitor Vieira Dourado, Manaus, Brazil; r Instituto Leônidas e Maria Deane, Fiocruz, Manaus, Brazil; s University of Texas Medical Branch, Galveston, TX, USA; t Laboratory of Parasitic Diseases, Oswaldo Cruz Institute, Fiocruz, Rio de Janeiro, Brazil; u Global Health and Tropical Medicine, Institute of Hygiene and Tropical Medicine, NOVA University of Lisbon, Lisbon, Portugal; v Unit of Leishmaniasis and Malaria, Instituto de Medicina Tropical Alexander von Humboldt, Universidad Peruana Cayetano Heredia, Lima, Peru; w Central Department of Microbiology, Tribhuvan University, Kirtipur, Nepal; x Department of Infectious Diseases, Kasturba Medical College, and Manipal Center for Infectious Diseases, Prasanna School of Public Health, Manipal Academy of Higher Education, Manipal, India; y Department of Parasitology, Faculty of Medicine, University of Indonesia, Jakarta, Indonesia; z Oxford University Clinical Research Unit, Hospital for Tropical Diseases, Ho Chi Minh City, Viet Nam; aa WWARN, Oxford, UK; ab Infectious Diseases Data Observatory (IDDO), Oxford, UK; ac General and Subspecialty Medicine, Grampians Health—Ballarat, Ballarat, VIC, Australia

## Abstract

**Background:**

Primaquine radical cure is used to treat dormant liver-stage parasites and prevent relapsing *Plasmodium vivax* malaria but is limited by concerns of haemolysis. We undertook a systematic review and individual patient data meta-analysis to investigate the haematological safety of different primaquine regimens for *P vivax* radical cure.

**Methods:**

For this systematic review and individual patient data meta-analysis, we searched MEDLINE, Web of Science, Embase, and Cochrane Central for prospective clinical studies of uncomplicated *P vivax* from endemic countries published between Jan 1, 2000, and June 8, 2023. We included studies if they had active follow-up of at least 28 days, if they included a treatment group with daily primaquine given over multiple days where primaquine was commenced within 3 days of schizontocidal treatment and was given alone or coadministered with chloroquine or one of four artemisinin-based combination therapies (ie, artemether–lumefantrine, artesunate–mefloquine, artesunate–amodiaquine, or dihydroartemisinin–piperaquine), and if they recorded haemoglobin or haematocrit concentrations on day 0. We excluded studies if they were on prevention, prophylaxis, or patients with severe malaria, or if data were extracted retrospectively from medical records outside of a planned trial. For the meta-analysis, we contacted the investigators of eligible trials to request individual patient data and we then pooled data that were made available by Aug 23, 2021. The main outcome was haemoglobin reduction of more than 25% to a concentration of less than 7 g/dL by day 14. Haemoglobin concentration changes between day 0 and days 2–3 and between day 0 and days 5–7 were assessed by mixed-effects linear regression for patients with glucose-6-phosphate dehydrogenase (G6PD) activity of (1) 30% or higher and (2) between 30% and less than 70%. The study was registered with PROSPERO, CRD42019154470 and CRD42022303680.

**Findings:**

Of 226 identified studies, 18 studies with patient-level data from 5462 patients from 15 countries were included in the analysis. A haemoglobin reduction of more than 25% to a concentration of less than 7 g/dL occurred in one (0·1%) of 1208 patients treated without primaquine, none of 893 patients treated with a low daily dose of primaquine (<0·375 mg/kg per day), five (0·3%) of 1464 patients treated with an intermediate daily dose (0·375 mg/kg per day to <0·75 mg/kg per day), and six (0·5%) of 1269 patients treated with a high daily dose (≥0·75 mg/kg per day). The covariate-adjusted mean estimated haemoglobin changes at days 2–3 were –0·6 g/dL (95% CI –0·7 to –0·5), –0·7 g/dL (–0·8 to –0·5), –0·6 g/dL (–0·7 to –0·4), and –0·5 g/dL (–0·7 to –0·4), respectively. In 51 patients with G6PD activity between 30% and less than 70%, the adjusted mean haemoglobin concentration on days 2–3 decreased as G6PD activity decreased; two patients in this group who were treated with a high daily dose of primaquine had a reduction of more than 25% to a concentration of less than 7 g/dL. 17 of 18 included studies had a low or unclear risk of bias.

**Interpretation:**

Treatment of patients with G6PD activity of 30% or higher with 0·25–0·5 mg/kg per day primaquine regimens and patients with G6PD activity of 70% or higher with 0·25–1 mg/kg per day regimens were associated with similar risks of haemolysis to those in patients treated without primaquine, supporting the safe use of primaquine radical cure at these doses.

**Funding:**

Australian National Health and Medical Research Council, Bill & Melinda Gates Foundation, and Medicines for Malaria Venture.

**Research in context**

**Evidence before this study**

We identified prospective studies and meta-analyses evaluating the effect of primaquine use and dose on the risk of haemolysis in patients with acute *Plasmodium vivax* malaria and normal glucose-6-phosphate dehydrogenase (G6PD) activity (ie, ≥30% of the adjusted median value for healthy male individuals), published in any language from database inception to June 8, 2023, using the terms “vivax”, “primaquine”, and “haemolysis” in MEDLINE, Web of Science, Embase, and Cochrane Central. An individual patient data meta-analysis including 1692 patients with normal G6PD activity found no difference in haemoglobin concentrations at the day of nadir for patients treated with or without primaquine, with no assessment of primaquine dose. A nested cohort study found that haemolysis after primaquine was greater in 33 female patients heterozygous for G6PD deficiency than in 198 patients with wild-type G6PD, with increased haemolysis in heterozygous patients after receiving 1 mg/kg per day of primaquine compared with 0·5 mg/kg per day.

**Added value of this study**

This individual patient data meta-analysis included data from 5462 patients with *P vivax* malaria with G6PD activity of 30% or higher, enrolled from 18 studies, and is the largest study to assess the risk of haemolysis with different primaquine daily doses. The results, co-published with an analysis of the effect of primaquine dose on treatment efficacy and tolerability, show similar mean haemoglobin concentrations at days 2–3 with and without primaquine use at a population level after adjustment for confounders. Severe haemolytic events were rare but more common after primaquine regimens at 1 mg/kg per day than after treatment without primaquine. Compared with patients with G6PD activity of 70% or higher, patients with G6PD activity between 30% and less than 70% had lower haemoglobin concentrations after treatment, with or without primaquine.

**Implications of all the available evidence**

Patients with G6PD activity of 30% or higher treated with primaquine regimens at 0·25 mg/kg per day and 0·5 mg/kg per day have a similar risk of haemolysis to those treated without primaquine, supporting the safe use of primaquine at these doses to prevent relapsing *P vivax* malaria.

## Introduction

*Plasmodium vivax* causes an estimated 4·9–14·3 million cases of malaria annually across 50 malaria-endemic countries in the Asia-Pacific region, Americas, and Africa.^1^, ^2^ *P vivax* malaria leads to substantial morbidity, particularly in children and pregnant women.^3^ *P vivax* forms dormant liver stages (ie, hypnozoites), which can reactivate weeks to months after the initial infection causing recurrent episodes of malaria (ie, relapses). Effective treatment and interruption of transmission requires a combination of antimalarial drugs targeting both the blood and liver-stage parasites (ie, radical cure). The 8-aminoquinolines—primaquine and tafenoquine—are the only effective antimalarials available to treat liver-stage hypnozoites. Primaquine, administered during a period of 7–14 days, has been used for more than 70 years, and remains the main treatment option in most endemic locations. Single-dose tafenoquine was licensed in 2018, and is now available in several malaria-endemic countries.^4^

The widespread use of 8-aminoquinolines is undermined by the risk of potentially severe haemolysis in individuals with glucose-6-phosphate dehydrogenase (G6PD) deficiency, which is present in up to 30% of individuals in some malaria-endemic locations.^5^ G6PD deficiency is an X-linked trait with more than 200 different genotypes that cause variable degrees of enzymopathy and vulnerability to haemolysis after oxidant stress.^6^ Affected hemizygous male individuals and homozygous female individuals generally have very low G6PD activity (<30% of the adjusted median value for healthy male individuals), whereas, in heterozygous female individuals, the enzymatic activity phenotype ranges between normal and deficient, due to random erythroid cell mosaicism of the trait (ie, lyonisation) during embryonic development.^7^

WHO recommends routine testing of G6PD deficiency and prescribing daily primaquine regimens only to individuals with normal G6PD activity (ie, ≥30%).^8^ Tafenoquine is licensed for individuals with G6PD activity of 70% or higher, a level that excludes most heterozygous females.^9^ Qualitative tests can identify individuals with activity of 30% or higher, whereas quantitative tests are required to identify individuals with activity of 70% or higher.^4^ In many malaria-endemic settings, the use of G6PD testing before antimalarial treatment is logistically and financially challenging. Consequently, many health-care providers either avoid the use of 8-aminoquinoline regimens, prescribe primaquine without G6PD testing, or administer primaquine at lower than recommended doses.^10^

The population and individual risks of haemolysis with different primaquine doses must be quantified and balanced against the haemolytic risk and consequent morbidity of recurrent parasitaemia in patients with relapsing malaria. We did a systematic review and individual patient data meta-analysis to investigate the effect of primaquine daily dose on the haemoglobin response in patients with vivax malaria and with G6PD activity of 30% or higher, in addition to determining the effect of G6PD activity (including activity between 30% and <70%) on haemoglobin response and how the risk of haemolysis is modified by primaquine dose.

## Methods

### Search strategy and selection criteria

For this systematic review and individual patient data meta-analysis, we searched for efficacy studies of patients with uncomplicated *P vivax* monoinfections published in any language between Jan 1, 2000, and Aug 23, 2021, and we updated the search in June, 2023, to include studies published until June 8, 2023, according to the PRISMA individual patient data statement (appendix pp 3–6). We searched MEDLINE, Web of Science, Embase, and Cochrane Central with the terms used in a previous review^11^ and listed in the appendix (p 7). We also reviewed the studies in the reference lists of the identified articles. We included studies if they were prospective with active follow-up of at least 28 days, if they included a treatment group with daily primaquine given over multiple days, where primaquine was commenced within 3 days of schizontocidal treatment, if primaquine was given alone or coadministered with chloroquine or one of four artemisinin-based combination therapies (ie, artemether–lumefantrine, artesunate–mefloquine, artesunate–amodiaquine, or dihydroartemisinin–piperaquine), and if they collected haemoglobin or haematocrit measurements on day 0. Reviews and animal studies; studies on prevention, prophylaxis, or patients with severe malaria; and studies where schizontocidal treatment was unsupervised or where data were extracted retrospectively from medical records outside of a planned trial were excluded. In June, 2023, we also did a post-hoc systematic review of the Scopus database, using the same search strategy and selection criteria.

Two reviewers (RJC and RNP) undertook the systematic review with discrepancies resolved through discussion. The protocol was registered with PROSPERO, CRD42019154470 and CRD42022303680.

### Data collation

Investigators of eligible studies were invited to share individual patient data, and also to contribute data from unpublished studies they had been involved with, by Aug 23, 2021. We did not request individual patient data for studies published between Aug 24, 2021, and June 8, 2023, due to the long time required to obtain approval and collate these data. Shared data were de-identified, collated, and standardised via the WorldWide Antimalarial Resistance Network repository.^12^ We excluded individual patient data if they were missing age, sex, baseline parasite density, baseline haemoglobin concentration, or primaquine daily mg/kg dosing data. We also excluded patients who had severe malaria or mixed species infection at baseline, who were pregnant, who were given adjunctive antimalarials within 14 days of starting treatment, or who were not known to have G6PD activity of 30% or higher.

Additional criteria identified studies that were eligible for a complementary prespecified analysis of the effect of G6PD activity on primaquine-associated haemolysis: studies were excluded if they did not report quantitative G6PD activity measurements by spectrophotometry or if the adjusted male median G6PD activity was unavailable and could not be calculated.

Shared data were obtained according to ethical approvals from the country of origin and original study. The data were anonymised and cannot be linked to individuals. As such, our analysis did not require additional ethics approval according to the guidelines of the Oxford Central University Research Ethics Committee.

### Outcomes

The primary outcome was haemoglobin reduction of more than 25% to a concentration of less than 7 g/dL between day 0 and days 1–14, reflecting a substantial decrease to a clinically relevant threshold. Secondary outcomes were (1) the maximum absolute change in haemoglobin concentration between day 0 and days 2–3 (expected day of nadir haemoglobin concentration^13^); (2) the maximum absolute change in haemoglobin concentration between day 0 and days 5–7 (high sampling rate); (3) a composite outcome of the presence of any of the following: post-treatment reduction in haemoglobin concentration to less than 5 g/dL or absolute decrease in haemoglobin concentration of more than 5 g/dL between day 0 and days 1–14, renal failure requiring dialysis between day 1 and day 28, blood transfusion between day 1 and day 28, or death between day 1 and day 28; and (4) the development of anaemia by days 2–3 or separately by days 5–7. Analyses were undertaken for individuals with G6PD activity of 30% or higher, with subgroup analyses undertaken in individuals with intermediate or normal G6PD activity by quantitative testing. In individuals with data on quantitative G6PD activity, additional outcomes included the lowest haemoglobin concentration on days 2–3 and on days 5–7.

### Data analysis

The daily mg/kg dose of primaquine was calculated from the number of tablets or the mg doses administered to each individual, or if unavailable, from the planned dosing regimen. The daily primaquine dose was defined as low (<0·375 mg base per kg per day), intermediate (≥0·375 mg base per kg per day to <0·75 mg base per kg per day), and high (≥0·75 mg base per kg per day), reflecting the spread of dosing around the two recommended daily dosing regimens (0·25 mg base per kg per day and 0·5 mg base per kg per day) and a proposed higher daily dosing regimen (1 mg base per kg per day).^14^

Anaemia was defined as mild (haemoglobin concentrations ≥8 g/dL to <11 g/dL), moderate (≥5 g/dL to <8 g/dL), or severe (<5 g/dL).^15^ If data on haemoglobin were unavailable, haematocrit was converted to haemoglobin concentration using the following formula: haemoglobin (g/dL)=(haematocrit [%] – 5·62)/2·60.^16^ G6PD activity was defined as deficient (<30% activity or a positive qualitative test), normal (≥30% activity or a negative qualitative test), or unknown (no G6PD testing undertaken). Quantitative G6PD activity was categorised as deficient (<30%), intermediate (30% to <70%), and normal (≥70%; appendix p 8).

The number and proportion of patients with the primary and the composite secondary outcome were calculated by primaquine dose groups. The population level profile of haemoglobin over time (days 0–42, to account for the acute fall and initial recovery) in patients treated with and without primaquine was estimated using a linear mixed-effects model with non-linear terms for time (appendix pp 8–9). Linear mixed-effects regression analyses were done to investigate the association between daily primaquine dose and change in haemoglobin concentrations between day 0 and the lowest haemoglobin concentration on days 2–3 and on days 5–7. In patients with baseline haemoglobin concentration of 11 g/dL or higher, the association between daily primaquine dose and the development of anaemia on days 2–3 was investigated using generalised estimating equation Poisson models with clustering by study site, exchangeable correlation structure, and robust standard error estimates, adjusting for age category, sex, log_10_ baseline parasite density, and baseline haemoglobin concentration (appendix p 8). A sensitivity analysis restricted the analyses to patients for whom the actual primaquine daily dose administered was known.

In patients with recorded quantitative G6PD activity, outcomes were described for those with intermediate and normal G6PD activity. Linear mixed-effects regression was undertaken to investigate whether G6PD activity was associated with haemoglobin concentrations at days 2–3 and at days 5–7 and whether primaquine daily dose was an effect modifier (appendix p 8). A sensitivity analysis restricted the analyses to patients with G6PD activity recorded at presentation of their acute malaria episode. Risk of bias assessments including assessment for within study bias, inclusion bias, and between study heterogeneity are described in the appendix (p 8). Assessment of publication bias was not done because the objective of the original studies did not align with our primary research question. Analyses were undertaken in R (version 4.1.3) and Stata (version 17) according to an a-priori statistical analysis plan.^17^

### Role of the funding source

The funders of the study had no role in study design, data collection, data analysis, data interpretation, or writing of the report.

## Results

**Figure 1:**
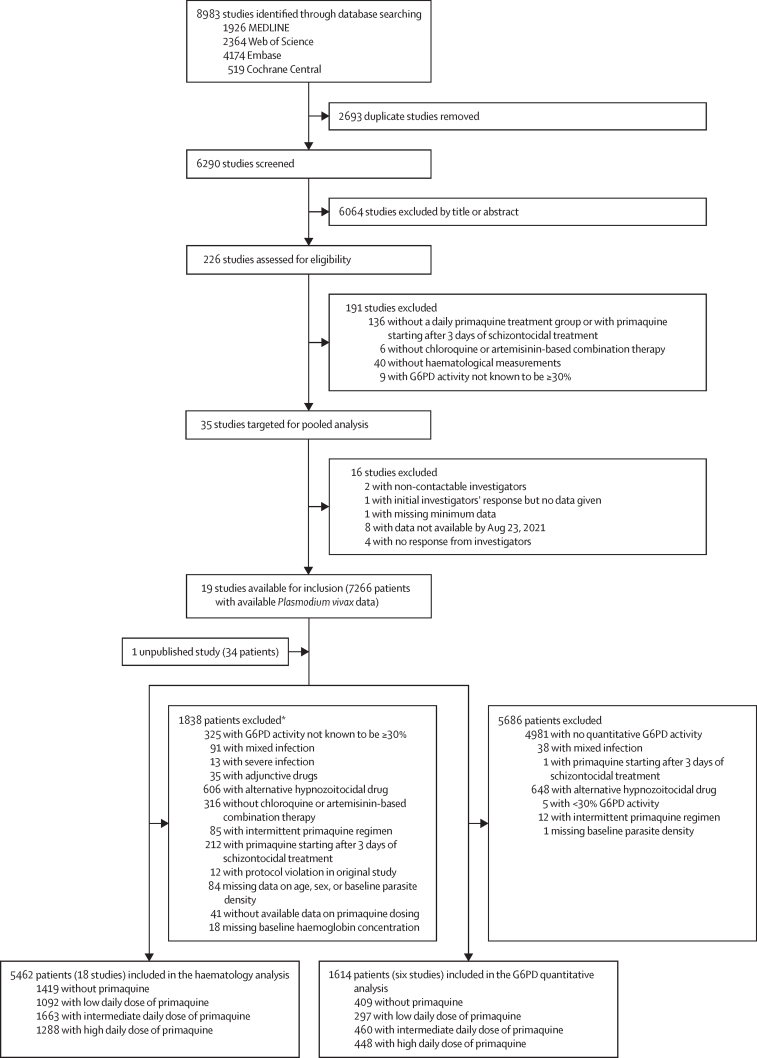
Study selection Patients in the G6PD quantitative analysis were a subgroup of patients in the overall haematology analysis. G6PD=glucose-6-phosphate dehydrogenase. *Includes all patients from two studies excluded on patient-level factors (appendix p 14) and individual patients from additional studies.

Between Jan 1, 2000, and June 8, 2023, we identified 8983 studies via database searching (figure 1). After removing 2693 duplicates and excluding 6064 studies in the title and abstract screening, we assessed 226 *P vivax* efficacy studies for eligibility, of which 35 met the inclusion criteria. Investigators from 19 (54·3%) studies shared individual patient data on 7266 patients with vivax malaria at presentation. An additional unpublished study with 34 patients was shared. 1838 patients were excluded, resulting in 5462 patients with G6PD activity of 30% or higher from 18 studies (one unpublished) and 15 countries being included in the haematological analysis (figure 1; appendix pp 10–16).^14^, ^18^, ^19^, ^20^, ^21^, ^22^, ^23^, ^24^, ^25^, ^26^, ^27^, ^28^, ^29^, ^30^, ^31^, ^32^, ^33^ In the post-hoc systematic review in Scopus, we did not identify additional eligible studies.

The median patients' age was 19·0 years (IQR 12·0–30·0); the majority of patients were male (3498 [64·0%]) and from the Asia-Pacific region (4097 [75·0%]; table 1). The low daily dose of primaquine was administered to 1092 (20·0%) patients, the intermediate daily dose to 1663 (30·4%) patients, and the high daily dose to 1288 (23·6%) patients; 1419 (26·0%) patients were treated without primaquine. A smaller proportion of patients treated with a low daily dose of primaquine started primaquine on day 0 and were given dihydroartemisinin–piperaquine, compared with patients treated with an intermediate and a high daily dose of primaquine (table 1). Included studies enrolled patients more recently and had longer patient follow-up than studies that were eligible for inclusion but not available (appendix p 17). Of the 18 included studies, 14 (one unpublished; 77·8%) were randomised controlled trials,^14^, ^18^, ^19^, ^21^, ^22^, ^25^, ^26^, ^27^, ^29^, ^30^, ^31^, ^32^, ^33^ four (22·2%) were non-randomised clinical efficacy studies,^20^, ^23^, ^24^, ^28^ and nine (one unpublished; 50·0%) compared treatments with and without primaquine.^14^, ^21^, ^25^, ^26^, ^27^, ^30^, ^32^, ^33^ 17 (one unpublished; 94·4%) of 18 included studies had low or unclear risk of bias (appendix pp 18–19).^14^, ^18^, ^19^, ^20^, ^21^, ^22^, ^23^, ^24^, ^25^, ^26^, ^27^, ^29^, ^30^, ^31^, ^32^, ^33^

**Table 1:** Demographic and baseline characteristics of patients with glucose-6-phosphate dehydrogenase activity of 30% or higher

	**Overall (n=5462)**	**Primaquine dose**			
		No primaquine (n=1419)	Low daily dose (<0·375 mg/kg per day; n=1092)	Intermediate daily dose (≥0·375 mg/kg per day to <0·75 mg/kg per day; n=1663)	High daily dose (≥0·75 mg/kg per day; n=1288)
**Sex**					
Male	3498 (64·0%)	888 (62·6%)	701 (64·2%)	1102 (66·3%)	807 (62·7%)
Female	1964 (36·0%)	531 (37·4%)	391 (35·8%)	561 (33·7%)	481 (37·3%)
**Age**					
Median, years	19·0 (12·0–30·0)	19·0 (12·0–30·0)	24·0 (15·8–37·0)	18·0 (11·8–29·0)	17·0 (10·8–26·0)
<5 years	329 (6·0%)	86 (6·1%)	68 (6·2%)	93 (5·6%)	82 (6·4%)
5–14 years	1537 (28·1%)	385 (27·1%)	185 (16·9%)	510 (30·7%)	457 (35·5%)
≥15 years	3596 (65·8%)	948 (66·8%)	839 (76·8%)	1060 (63·7%)	749 (58·2%)
**Enrolment variables**					
Weight, kg	50·0 (30·0–59·0)	50·0 (31·9–60·0)	55·0 (45·0–64·2)	48·0 (29·0–57·0)	45·7 (26·0–55·0)
Malnutrition*	86/401 (21·4%)	24/99 (24·2%)	16/85 (18·8%)	28/121 (23·1%)	18/96 (18·8%)
Presence or recent history of fever	4850/5189 (93·5%)	1267/1344 (94·3%)	956/982 (97·4%)	1457/1577 (92·4%)	1170/1286 (91·0%)
Parasitaemia, parasites per mL	3850·0 (1281·5–10 299·0)	4348·1 (1696·3–10 722·2)	3356·5 (1317·0–7895·5)	3744·0 (1017·0–10 676·0)	4483·3 (1165·3–12 500·0)
Haemoglobin, g/dL	12·7 (1·8)	12·6 (1·8)	12·6 (2·0)	12·7 (1·8)	12·8 (1·8)
**Schizontocidal treatment**†					
Chloroquine	3711/5377 (69·0%)	1101 (77·6%)	900 (82·4%)	1012/1620 (62·5%)	698/1246 (56·0%)
Artemether–lumefantrine	233/5377 (4·3%)	122 (8·6%)	94 (8·6%)	10/1620 (0·6%)	7/1246 (0·6%)
Artesunate–amodiaquine	51/5377 (0·9%)	0	51 (4·7%)	0/1620	0/1246
Artesunate–mefloquine	0/5377	0	0	0/1620	0/1246
Dihydroartemisinin–piperaquine	1382/5377 (25·7%)	196 (13·8%)	47 (4·3%)	598/1620 (36·9%)	541/1246 (43·4%)
**Primaquine dosing**					
Total dose, mg/kg	6·8 (4·2–7·5)	..	3·6 (3·1–4·2)	7·0 (6·5–7·7)	7·2 (6·7–7·9)
Daily dose, mg/kg	0·5 (0·3–1·0)	..	0·3 (0·2–0·3)	0·5 (0·5–0·6)	1·0 (1·0–1·1)
**Primaquine dose derived from**					
Actual dosing	3414/4043 (84·4%)	..	723 (66·2%)	1450 (87·2%)	1241 (96·4%)
Protocol dosing	629/4043 (15·6%)	..	369 (33·8%)	213 (12·8%)	47 (3·6%)
**Primaquine start day**					
Day 0	3450/4043 (85·3%)	..	531 (48·6%)	1632 (98·1%)	1287 (99·9%)
Day 1	264/4043 (6·5%)	..	260 (23·8%)	4 (0·2%)	0 (0·0%)
Day 2	329/4043 (8·1%)	..	301 (27·6%)	27 (1·6%)	1 (0·1%)
**Primaquine duration**					
7–10 days	1417/4043 (35·0%)	..	2 (0·2%)	156 (9·4%)	1259 (97·7%)
14 days	2626/4043 (65·0%)	..	1090 (99·8%)	1507 (90·6%)	29 (2·3%)
**Relapse periodicity**‡					
Low	2490 (45·6%)	792 (55·8%)	780 (71·4%)	505 (30·4%)	413 (32·1%)
High	2972 (54·4%)	627 (44·2%)	312 (28·6%)	1158 (69·6%)	875 (67·9%)
**Region**					
Africa	974 (17·8%)	315 (22·2%)	196 (17·9%)	233 (14·0%)	230 (17·9%)
Americas	391 (7·2%)	119 (8·4%)	180 (16·5%)	88 (5·3%)	4 (0·3%)
Asia-Pacific	4097 (75·0%)	985 (69·4%)	716 (65·6%)	1342 (80·7%)	1054 (81·8%)

Data are n (%), median (IQR), mean (SD), or n/N (%). Data were not available for 117 patients on weight.

* The nutritional status of children younger than 5 years was calculated as a weight-for-age Z score, using the igrowup package developed by WHO,^34^ with children with Z scores smaller than −2 classified as having malnutrition and malnutrition status considered missing if Z scores were smaller than −6 or larger than 6.

† 85 patients received primaquine alone without schizontocidal treatment.

‡ Relapse periodicity (ie, the time from initial infection to vivax relapse) was classified by geographical region as low or high, according to a median interval from first illness to relapse of more than 47 days or 47 days or less, respectively.

Overall, 12 (0·2%) of 4834 patients with G6PD activity of 30% or higher had a reduction in haemoglobin concentration larger than 25% to a concentration of less than 7 g/dL from baseline to days 1–14 (one [0·4%] of 273 patients aged <5 years, seven [0·5%] of 1405 patients aged 5 years to <15 years, and four [0·1%] of 3156 patients aged ≥15 years). This outcome was observed in one (0·1%) of 1208 patients treated without primaquine, none of 893 patients treated with a low daily dose of primaquine, five (0·3%) of 1464 patients treated with an intermediate daily dose, and six (0·5%) of 1269 patients treated with a high daily dose (table 2). Eight (66·7%) of 12 patients were female; however, quantitative G6PD activities were only available for three (25·0%) patients, who had 37·4%, 61·5%, and 94·8% activity (appendix p 20).

**Table 2:** Haematological safety outcomes in patients with glucose-6-phosphate dehydrogenase activity of 30% or higher

	**No primaquine**	**Low daily dose primaquine (<0·375 mg/kg per day)**	**Intermediate daily dose primaquine (≥0·375 mg/kg per day to <0·75 mg/kg per day)**	**High daily dose primaquine (≥0·75 mg/kg per day)**
**>25% reduction in haemoglobin concentration to <7 g/dL**				
Percentage (95% CI)	0·1% (0·0 to 0·5)	0·0% (0·0 to 0·4)	0·3% (0·1 to 0·8)	0·5% (0·2 to 1·0)
n/N	1/1208	0/893	5/1464	6/1269
Absolute change in haemoglobin concentration between day 0 and days 2–3 (g/dL), unadjusted*				
Mean (SD)	−0·5 (1·0)	−0·5 (0·9)	−0·7 (1·1)	−0·7 (1·1)
Range	−6·0 to 4·0	−4·6 to 6·0	−5·5 to 7·5	−5·7 to 4·3
N	1146	831	1392	1209
Absolute change in haemoglobin concentration between day 0 and days 5–7 (g/dL), unadjusted*				
Mean (SD)	−0·2 (1·0)	−0·2 (0·9)	−0·4 (1·2)	−0·5 (1·4)
Range	−4·9 to 5·2	−3·5 to 6·0	−6·1 to 8·2	−6·2 to 3·5
N	1130	833	1348	1179
**Composite measure of haemoglobin change**†				
Percentage (95% CI)	0·1% (0·0 to 0·5)	0·0% (0·0 to 0·4)	0·5% (0·2 to 1·1)	0·6% (0·3 to 1·2)
n/N	1/1208	0/893	8/1464	8/1269

* Negative values for absolute change indicate a decrease compared with baseline, whereas positive values indicate an increase.

† Composite measure of haemoglobin change is a composite indicator of a post-treatment reduction in haemoglobin concentration to less than 5 g/dL or an absolute decrease in haemoglobin concentration of more than 5 g/dL from day 0 to days 1–14, renal failure needing dialysis, blood transfusion, or death between day 1 and day 28. The 95% CIs are binomial exact confidence intervals.

**Figure 2:**
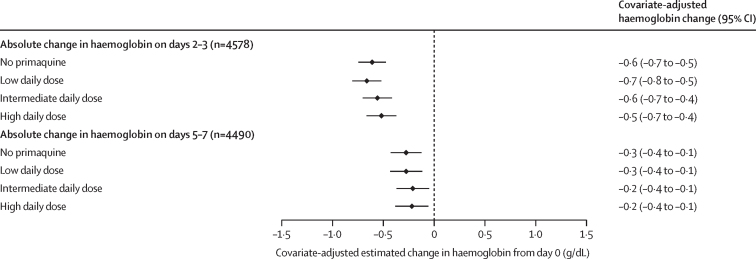
Covariate-adjusted estimated mean change in haemoglobin from baseline to days 2–3 or days 5–7 by primaquine treatment regimen in patients with G6PD activity of 30% or higher Linear mixed-effects models for change between day 0 and days 2–3 and separately for change between day 0 and days 5–7 were adjusted for baseline haemoglobin concentration, age, sex, and log_10_ baseline parasite density, with random effects for study site in each model. Adjusted haemoglobin absolute change values were estimated using mean values for baseline haemoglobin concentration, age, sex, and log_10_ baseline parasite density. G6PD=glucose-6-phosphate dehydrogenase.

In patients with G6PD activity of 30% or higher, the haemoglobin profiles between patients treated with or without primaquine were similar on days 2–3 (appendix p 21). The unadjusted mean change in haemoglobin on days 2–3 compared with day 0 was –0·5 g/dL (SD 1·0) in patients treated without primaquine, –0·5 g/dL (0·9) in those treated with a low daily dose of primaquine, –0·7 g/dL (1·1) in those treated with an intermediate daily dose, and –0·7 g/dL (1·1) in those treated with a high daily dose (table 2). After adjusting for confounders, including haemoglobin concentration on day 0, there was no difference between primaquine dose groups in the estimated covariate-adjusted mean difference in haemoglobin from baseline to either days 2–3 or days 5–7 (figure 2); results were similar in sensitivity analyses (appendix p 22).

The secondary composite outcome measure of haemolysis (reduction in haemoglobin concentration to <5 g/dL or absolute decrease of >5 g/dL, renal failure requiring dialysis, blood transfusion, or death) occurred in one (0·1%) patient treated without primaquine, none with a low daily dose of primaquine, eight (0·5%) with an intermediate daily dose, and eight (0·6%) with a high daily dose (table 2).

The unadjusted risk of developing anaemia of any severity on days 2–3 in patients with a baseline haemoglobin concentration of 11 g/dL or higher was 9·3% (89/957) with no primaquine, 7·0% (49/701) with a low daily dose of primaquine, 15·6% (186/1189) with an intermediate daily dose, and 17·3% (176/1018) with a high daily dose (appendix p 23). A higher unadjusted risk of developing anaemia with the intermediate and high daily doses of primaquine was also apparent on days 5–7 (appendix p 23). After adjusting for potential confounders, the risk of developing anaemia of any severity on days 2–3 or on days 5–7 in patients receiving any dose of primaquine was similar to that observed in patients not treated with primaquine (appendix p 23).

Six (33·3%) of 18 studies eligible for the overall haematology analysis recorded quantitative G6PD activity,^14^, ^21^, ^23^, ^29^, ^30^, ^32^ with data available for 1614 patients (figure 1; appendix pp 10–11). 51 (3·2%) of 1614 patients had intermediate G6PD activity (30% to <70%) and 1563 (96·8%) had normal activity (≥70%; appendix pp 24–25).

**Figure 3:**
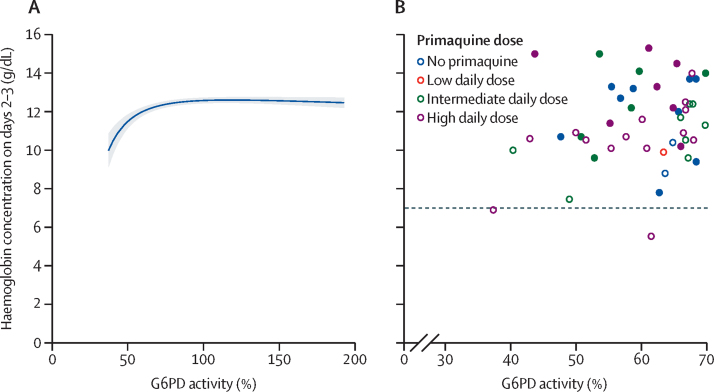
G6PD activity versus haemoglobin concentration on days 2–3 (A) Modelled association in patients with quantitative G6PD activity of 30% or higher. Shaded region shows 95% CIs. The linear mixed-effects model with fractional polynomial terms for the relationship between G6PD activity and haemoglobin on days 2–3 was adjusted for day 0 haemoglobin, age, sex, log_10_ baseline parasite density, and primaquine treatment regimen, with interaction terms between G6PD activity and primaquine groups, and random effects for study site. (B) Observed day 2–3 haemoglobin concentrations by G6PD activity between 30% and less than 70%. Closed circles show male patients and open circles show female patients. The dashed line indicates the clinically relevant threshold of 7 g/dL. G6PD=glucose-6-phosphate dehydrogenase.

After adjusting for confounders, the mean haemoglobin concentration on days 2–3 and on days 5–7 was positively associated with G6PD activity between 30% and less than 70% (figure 3; appendix pp 26–27). The number of patients with G6PD activity between 30% and less than 70% was too small to assess the association between primaquine dose and haemoglobin concentrations on days 2–3 or on days 5–7; however, in patients with G6PD activity of 70% or higher, the association between G6PD activity and haemoglobin concentration on days 2–3 or on days 5–7 was not modified by primaquine (appendix pp 28–29). Sensitivity analyses restricting inclusion to patients with G6PD activity of 30% or higher measured at the time of the acute malaria episode showed similar results to the analysis including patients with G6PD acitivity of 30% or higher measured at any time (appendix pp 30–31). The positive association between haemoglobin concentrations on days 2–3 and G6PD activity between 30% and less than 70% was also present in the subgroup of patients treated without primaquine (appendix p 32).

Of patients with quantitative G6PD activity measurements, no patients treated without primaquine or with low or intermediate daily doses of primaquine had a haemoglobin reduction of more than 25% to a concentration of less than 7 g/dL. Two (9·1%) of 22 patients (both female) with intermediate G6PD activity who were treated with a high daily dose of primaquine had such a reduction: one on day 3 (37% G6PD activity, from 11·6 g/dL to 6·9 g/dL) and one on day 5 (62% G6PD activity, from 8·8 g/dL to 5·5 g/dL). One female patient (0·2%) of 426 patients with a G6PD activity of 95% who was treated with a high daily dose of primaquine had a decrease in haemoglobin concentration from 9·8 g/dL to 6·3 g/dL on day 5 (figure 3; appendix pp 22, 26, 28–29). The composite haemolytic outcome did not occur in any patients treated with a low daily dose of primaquine or without primaquine. In patients with intermediate G6PD activity, the composite outcome did not occur in patients treated with an intermediate daily dose of primaquine, but occurred in one (4·5%) of 22 patients treated with a high daily dose. In patients with a G6PD activity of 70% or higher, the composite outcome occurred in one (0·2%) of 467 patients treated with an intermediate daily dose and one (0·2%) of 426 patients treated with a high daily dose (appendix pp 22, 28–29).

## Discussion

The potential for haemolysis in patients with *P vivax* malaria treated with 8-aminoquinolines remains a key barrier to effective implementation of radical cure. Our individual patient data meta-analysis describes the haematological effects of different primaquine dosing regimens in different endemic settings, highlighting that in patients with G6PD activity of 30% or higher both the 0·25 mg/kg and 0·5 mg/kg daily doses were well tolerated and in patients with G6PD activity of 70% or higher all daily dose regimens had acceptable haematological profiles.

Irrespective of the antimalarials administered, all patients with vivax malaria have infection-induced haemolysis. At a population level, haemoglobin concentration reaches a nadir on days 2–3 after initiating treatment, before a gradual increase, returning to baseline concentration at about 1–2 weeks.^13^ The initial change in haemoglobin concentration is strongly associated with the concentration at baseline—a higher concentration at baseline is associated with a greater initial decrease.^13^ In the current analysis, the initial decrease and subsequent increase in haemoglobin concentration is mirrored in individuals who received primaquine at any dose. In patients with G6PD activity of 30% or higher, there was no association between primaquine dose and the absolute decrease in haemoglobin concentration.

Higher total primaquine doses are more effective than lower doses,^35^, ^36^ and are generally standard practice in countries where G6PD testing is routinely available.^37^ In areas where G6PD testing is not available, most malaria-endemic countries adopt a primaquine regimen at a lower dose (ie, 3·5 mg/kg total dose) administered during 7 days or 14 days.^38^ However, this low-dose regimen comes at the cost of an increased risk of relapsing infections and a cumulative risk of parasite-induced anaemia.^39^ In patients with G6PD activity of 30% or higher, the risk of severe haemolytic events was rare and occurred in patients treated with and without primaquine. Most events occurred after treatment with the highest daily primaquine dose (1 mg/kg per day), with six (0·5%) of 1269 patients' haemoglobin concentrations falling by more than 25% to a concentration of less than 7 g/dL. Owing to the small number of events, we were unable to adjust for confounders, such as baseline haemoglobin concentration, to compare this risk between dosing groups. Although the unadjusted proportion of patients developing anaemia (haemoglobin concentration <11 g/dL) was similarly higher in patients treated with 0·5 mg/kg per day and 1 mg/kg per day of primaquine compared with those who received no primaquine or 0·25 mg/kg per day, the risk of developing anaemia was similar across all groups after adjusting for confounders.

Unlike the majority of G6PD-deficient male individuals who have a G6PD activity of less then 30%, heterozygous female individuals can have a spectrum of activities, with approximately 60% of them having an intermediate activity (30 to <70%).^40^ We identified 51 patients with intermediate G6PD activity, of whom two female patients had a decrease in haemoglobin concentration of more than 25% to a concentration of less than 7 g/dL after treatment with 1 mg/kg per day primaquine (one of whom had a blood transfusion). A previous nested cohort study of heterozygous female individuals, whose data were included in this study, found an increased risk of haemolysis with 1 mg/kg per day of primaquine compared with 0·5 mg/kg per day.^41^ In patients known to have a G6PD activity of 70% or higher, there was no association between primaquine dose and haemolysis, with no meaningful difference in population means or in the number of patients with severe events. Although a threshold of 30% activity might be appropriate in patients treated with ≤0·5 mg/kg per day, a higher threshold of activity (≥70%) might be warranted in patients treated with the higher 1 mg/kg daily dose. The higher threshold would be similar to the threshold currently recommended for tafenoquine by the manufacturer (GSK, Abbotsford, VIC, Australia),^42^ which was instituted given the drug's long half-life and the inability to intervene and cease treatment if haemolysis occurred. Alternatively, increased pharmacovigilance with additional patient reviews on days 2–3 to identify patients at increased risk of severe haemolysis could be considered for 1 mg/kg per day primaquine regimens.

Although affordable point-of-care G6PD tests have been developed, these have not passed WHO prequalification to date, and thus their use in clinical practice remains limited. However, a quantitative point-of-care test to measure G6PD activity (STANDARD G6PD test),^43^ which was developed by SD Biosensor (Suwon, South Korea) in 2018, has received interim approval from the Expert Review Panel for Diagnostics,^44^ allowing for increased use to identify patients with different degrees of G6PD deficiency, so that radical cure regimens can be adjusted accordingly. In patients with less than 30% G6PD activity, treatment with 0·75 mg/kg primaquine weekly for 8 weeks is recommended by WHO, whereas patients with an activity of 30% or higher are given daily primaquine at 0·25 mg/kg per day to 0·5 mg/kg per day for 7 days or 14 days.^8^ Patients with G6PD activity of 70% or higher could potentially be treated with tafenoquine. The point-of-care test has now been implemented in eight countries at a subnational or national level (Bangladesh, Brazil, Cambodia, Laos, Myanmar, Solomon Islands, Thailand, and Viet Nam).^45^ Some barriers to its implementation in remote and low-resourced locations remain, including a need for training and a high cost for the devices and test strips.^46^

Our study has several limitations, including measurement of haemoglobin concentrations on differing days of follow-up between studies and the potential for unmeasured confounding. Data were available from 54% of eligible studies, with 89% of them published between 2009 and 2021. Four studies published after Aug 23, 2021 were not included because of the time required to collate individual patient data. Data on the actual daily dose of primaquine administered were available only for 86% of patients, although results did not differ in a sensitivity analysis restricted to these patients. Although the population effect of different primaquine doses on changes in haemoglobin concentration and the risk of anaemia could be assessed after adjusting for confounders, this adjustment was not possible for severe haemolytic events because of their small number, making interpretation of these results more difficult. Similarly, although quantitative G6PD activity was available for 30% of all patients, only 51 of these patients had intermediate G6PD activity, limiting the interpretation of the effect of different primaquine doses and confounder-adjusted analyses of the risk of haemolysis in this group of patients compared with those with a normal G6PD activity. Different G6PD genotypes lead to variable risks of haemolysis and genotypes were not known for most patients, thus preventing further assessment. Finally, two^23^, ^29^ of six^14^, ^21^, ^23^, ^29^, ^30^, ^32^ studies that measured quantitative G6PD activity assessed activity during convalescence rather than the acute malaria episode. A reduction in G6PD activity occurs across the lifespan of red blood cells and preferential destruction of older red blood cells occurs during acute malaria, which might elevate G6PD activity. This potential variation in G6PD activity might lead to bias when pooling activities measured during acute and steady state periods.^47^ Reassuringly, results were similar in sensitivity analyses restricted to patients with G6PD activity measured during acute malaria.

Additional data are required to quantify the haemolytic risk in patients with intermediate activity treated with a high daily dose of primaquine (1 mg/kg per day). Furthermore, operational feasibility studies are needed to assess the risk of severe haemolysis in routine clinical practice associated with different primaquine treatment regimens and the inevitable errors that will occur in implementing point-of-care G6PD testing. Patients with G6PD activity of less than 30% were not included in the current study; however, further studies are underway to establish the safety and efficacy of suitable treatment regimens in this population.^48^

In summary, compared with patients not treated with primaquine, the risk of severe haemolytic events with 0·25 mg/kg per day and 0·5 mg/kg per day primaquine dosing was low in patients with G6PD activity of 30% or higher, and with 1 mg/kg per day primaquine in patients with an activity of 70% or higher. The risk of severe haemolysis in patients with activity between 30% and less than 70% treated with 1 mg/kg per day primaquine remains unclear. These reassuring safety findings, in combination with evidence of the benefit of increased total mg/kg primaquine doses,^36^ pave the way for the widespread implementation of more efficacious primaquine regimens to reduce morbidity associated with vivax malaria.

## Data sharing

De-identified participant data used in this analysis are available for access via the WWARN website (https://www.wwarn.org/). Requests for access will be reviewed by a data access committee to ensure that use of data protects the interests of the participants and researchers according to the terms of ethics approval and principles of equitable data sharing. Requests can be submitted by email to malariaDAC@iddo.org via the data access form available at https://www.wwarn.org/working-together/sharing-accessing-data/accessing-data. WWARN is registered with the Registry of Research Data Repositories (https://www.re3data.org/).

## Declaration of interests

JAG and GCKWK are former employees of GSK and hold shares in GSK and AstraZeneca. GCKWK reports travel support from AstraZeneca. JKB reports institutional research funding from Medicines for Malaria Venture, GSK, Wellcome Trust, and Sanaria; participation on the US National Institutes of Health data safety monitoring board; and membership of the editorial board of *Travel Medicine and Infectious Disease* and the guidelines development group for malaria control and elimination, Global Malaria Programme, WHO. JKB, RNP, and RJC report contributions to Up-to-Date. All other authors declare no competing interests.
