# Supplementary material for: Primaquine dose and the risk of haemolysis in patients with uncomplicated Plasmodium vivax malaria: a systematic review and individual patient data meta-analysis
Source: Lancet Infect Dis. Author manuscript; Available in PMC 2024 Feb 16. (PMC7615565; doi:10.1016/S1473-3099(23)00431-0)
Supplement: supplement [file NIHMS1953839-supplement-supplement.pdf]

THE LANCET
Infectious Diseases

Supplementary appendix

This appendix formed part of the original submission and has been peer reviewed. We post it as supplied by the authors.

Supplement to: Rajasekhar M, Simpson JA, Ley B, et al. Primaquine dose and the risk of haemolysis in patients with uncomplicated *Plasmodium vivax* malaria: a systematic review and individual patient data meta-analysis. *Lancet Infect Dis* 2023; published online Sept 22. https://doi.org/10.1016/S1473-3099(23)00431-0.

APPENDIX

Rajasekhar M, *et al*, Primaquine dose and the risk of haemolysis in patients with uncomplicated *Plasmodium vivax* malaria: a systematic review and individual patient data meta-analysis

	Page
**Checklist S1.** PRISMA-IPD	3
**Box S1.** Search Strategy	7
**Text S1.** Methodology supplement	8
**Figure S1.** Directed acyclic graph	9
**Table S1.** Studies included in analysis	10
**Table S2.** Study testing and inclusion criteria	11
**Table S3.** Study sites included in analysis	12
**Table S4.** Reasons for studies not being included in analysis	14
**Table S5.** Studies eligible for the analysis but not included	15
**Figure S2.** Location of study sites	16
**Table S6.** Comparison of baseline characteristics between included and eligible but not included studies	17
**Table S7.** Risk of bias assessment in randomised controlled studies	18
**Table S8.** Risk of bias assessment in single arm observational studies	19
**Table S9.** Patients experiencing primary or secondary haematological outcomes or moderate or severe anaemia	20
**Figure S3.** Haemoglobin profiles of patients with ≥30% G6PD activity treated with or without primaquine	21
**Table S10.** Sensitivity analyses of change in haemoglobin from day 0 to day 2-3 and day 0 to days 5-7	22
**Table S11.** Risk of and adjusted risk ratios for anaemia on day 2-3 and days 5-7 in patients with G6PD ≥30%	23
**Table S12.** Demographics and baseline characteristics for patients with G6PD activity ≥30% to <70%	24
**Table S13.** Demographics and baseline characteristics for patients with G6PD activity ≥70%	25
**Figure S4.** A) Model of G6PD activity compared with the haemoglobin on days 5-7 in patients with quantitative G6PD activity ≥30% and B) unadjusted G6PD activity versus haemoglobin from day 0 to day 5-7 in patients with G6PD activity ≥30% to <70%	26
**Figure S5.** Unadjusted plot of G6PD activity versus change in haemoglobin from day 0 to A) day 2-3 and B) days 5-7	27
**Table S14.** Haematological safety outcomes in patients with G6PD activity ≥30% to <70% and ≥70% by primaquine daily dose groups	28
**Table S15.** Haematological safety outcomes in patients with G6PD activity ≥30% to <70% for A) female and B male patients by primaquine daily dose groups	29

Figure S6. Sensitivity analysis restricting to G6PD activity measured during acute malaria episodes of A) the model of G6PD activity compared with the haemoglobin on day 2-3 and B) unadjusted G6PD activity versus change in haemoglobin from day 0 to day 2-3 30

Figure S7. Sensitivity analysis restricting to G6PD activity measured during acute malaria episodes of A) the model of G6PD activity compared with the haemoglobin on days 5-7 and B) unadjusted G6PD activity versus change in haemoglobin from day 0 to day 5-7 31

Figure S8. Model of G6PD activity compared with haemoglobin on day 2-3 in patients not treated with primaquine with quantitative G6PD activity ≥30% 32

References S1. Studies not included in analysis 33

Checklist S1. PRISMA-IPD Checklist of items to include when reporting a systematic review and meta-analysis of individual participant data (IPD)

PRISMA-IPD Section/topic	Item No	Checklist item	Reported on page
**Title**			
Title	1	Identify the report as a systematic review and meta-analysis of individual participant data.	1
**Abstract**			
Structured summary	2	Provide a structured summary including as applicable:	6
		**Background**: state research question and main objectives, with information on participants, interventions, comparators and outcomes.	
		**Methods**: report eligibility criteria; data sources including dates of last bibliographic search or elicitation, noting that IPD were sought; methods of assessing risk of bias.	
		**Results**: provide number and type of studies and participants identified and number (%) obtained; summary effect estimates for main outcomes (benefits and harms) with confidence intervals and measures of statistical heterogeneity. Describe the direction and size of summary effects in terms meaningful to those who would put findings into practice.	
		**Discussion:** state main strengths and limitations of the evidence, general interpretation of the results and any important implications.	
		**Other:** report primary funding source, registration number and registry name for the systematic review and IPD meta-analysis.	
**Introduction**			
Rationale	3	Describe the rationale for the review in the context of what is already known.	8-11
Objectives	4	Provide an explicit statement of the questions being addressed with reference, as applicable, to participants, interventions, comparisons, outcomes and study design (PICOS). Include any hypotheses that relate to particular types of participant-level subgroups.	11
**Methods**			
Protocol and registration	5	Indicate if a protocol exists and where it can be accessed. If available, provide registration information including registration number and registry name. Provide publication details, if applicable.	14
Eligibility criteria	6	Specify inclusion and exclusion criteria including those relating to participants, interventions, comparisons, outcomes, study design and characteristics (e.g. years when conducted, required minimum follow-up). Note whether these were applied at the study or individual level i.e. whether eligible participants were included (and ineligible participants excluded) from a study that included a wider population than specified by the review inclusion criteria. The rationale for criteria should be stated.	12-13
Identifying studies – information sources	7	Describe all methods of identifying published and unpublished studies including, as applicable: which bibliographic databases were searched with dates of coverage; details of any hand searching including of conference proceedings; use of study registers and agency or company databases; contact with the original research team and experts in the field; open adverts and surveys. Give the date of last search or elicitation.	12
Identifying studies – search	8	Present the full electronic search strategy for at least one database, including any limits used, such that it could be repeated.	Appendix p7

Study selection processes	9	State the process for determining which studies were eligible for inclusion.	12
Data collection processes	10	Describe how IPD were requested, collected and managed, including any processes for querying and confirming data with investigators. If IPD were not sought from any eligible study, the reason for this should be stated (for each such study).	12
		If applicable, describe how any studies for which IPD were not available were dealt with. This should include whether, how and what aggregate data were sought or extracted from study reports and publications (such as extracting data independently in duplicate) and any processes for obtaining and confirming these data with investigators.	Appendix pp8, 17
Data items	11	Describe how the information and variables to be collected were chosen. List and define all study level and participant level data that were sought, including baseline and follow-up information. If applicable, describe methods of standardising or translating variables within the IPD datasets to ensure common scales or measurements across studies.	12, Refs 12, 13, 15
IPD integrity	A1	Describe what aspects of IPD were subject to data checking (such as sequence generation, data consistency and completeness, baseline imbalance) and how this was done.	Refs 12, 13
Risk of bias assessment in individual studies.	12	Describe methods used to assess risk of bias in the individual studies and whether this was applied separately for each outcome. If applicable, describe how findings of IPD checking were used to inform the assessment. Report if and how risk of bias assessment was used in any data synthesis.	Appendix p8
Specification of outcomes and effect measures	13	State all treatment comparisons of interests. State all outcomes addressed and define them in detail. State whether they were pre-specified for the review and, if applicable, whether they were primary/main or secondary/additional outcomes. Give the principal measures of effect (such as risk ratio, hazard ratio, difference in means) used for each outcome.	14-15, appendix p8
Synthesis methods	14	Describe the meta-analysis methods used to synthesise IPD. Specify any statistical methods and models used. Issues should include (but are not restricted to):<br>• Use of a one-stage or two-stage approach.<br>• How effect estimates were generated separately within each study and combined across studies (where applicable).<br>• Specification of one-stage models (where applicable) including how clustering of patients within studies was accounted for.<br>• Use of fixed or random effects models and any other model assumptions, such as proportional hazards.<br>• How (summary) survival curves were generated (where applicable).<br>• Methods for quantifying statistical heterogeneity (such as I^2 and t^2).<br>• How studies providing IPD and not providing IPD were analysed together (where applicable).<br>• How missing data within the IPD were dealt with (where applicable).	14-15, appendix p8
Exploration of variation in effects	A2	If applicable, describe any methods used to explore variation in effects by study or participant level characteristics (such as estimation of interactions between effect and covariates). State all participant-level characteristics that were analysed as potential effect modifiers, and whether these were pre-specified.	15, appendix p8
Risk of bias across studies	15	Specify any assessment of risk of bias relating to the accumulated body of evidence, including any pertaining to not obtaining IPD for particular studies, outcomes or other variables.	15, appendix pp8, 17

Additional analyses	16	Describe methods of any additional analyses, including sensitivity analyses. State which of these were pre-specified.	14-15, appendix p8
**Results**			
Study selection and IPD obtained	17	Give numbers of studies screened, assessed for eligibility, and included in the systematic review with reasons for exclusions at each stage. Indicate the number of studies and participants for which IPD were sought and for which IPD were obtained. For those studies where IPD were not available, give the numbers of studies and participants for which aggregate data were available. Report reasons for non-availability of IPD. Include a flow diagram.	16, Fig1, appendix pp10-15
Study characteristics	18	For each study, present information on key study and participant characteristics (such as description of interventions, numbers of participants, demographic data, unavailability of outcomes, funding source, and if applicable duration of follow-up). Provide (main) citations for each study. Where applicable, also report similar study characteristics for any studies not providing IPD.	Appendix pp10-13
IPD integrity	A3	Report any important issues identified in checking IPD or state that there were none.	16
Risk of bias within studies	19	Present data on risk of bias assessments. If applicable, describe whether data checking led to the up-weighting or down-weighting of these assessments. Consider how any potential bias impacts on the robustness of meta-analysis conclusions.	Appendix pp18-19
Results of individual studies	20	For each comparison and for each main outcome (benefit or harm), for each individual study report the number of eligible participants for which data were obtained and show simple summary data for each intervention group (including, where applicable, the number of events), effect estimates and confidence intervals. These may be tabulated or included on a forest plot.	16-19
Results of syntheses	21	Present summary effects for each meta-analysis undertaken, including confidence intervals and measures of statistical heterogeneity. State whether the analysis was pre-specified, and report the numbers of studies and participants and, where applicable, the number of events on which it is based.	16-19
		When exploring variation in effects due to patient or study characteristics, present summary interaction estimates for each characteristic examined, including confidence intervals and measures of statistical heterogeneity. State whether the analysis was pre-specified. State whether any interaction is consistent across trials.	
		Provide a description of the direction and size of effect in terms meaningful to those who would put findings into practice.	
Risk of bias across studies	22	Present results of any assessment of risk of bias relating to the accumulated body of evidence, including any pertaining to the availability and representativeness of available studies, outcomes or other variables.	Appendix p17
Additional analyses	23	Give results of any additional analyses (e.g. sensitivity analyses). If applicable, this should also include any analyses that incorporate aggregate data for studies that do not have IPD. If applicable, summarise the main meta-analysis results following the inclusion or exclusion of studies for which IPD were not available.	17-20, appendix pp21,30-31
**Discussion**			
Summary of evidence	24	Summarise the main findings, including the strength of evidence for each main outcome.	20-24

Strengths and limitations	25	Discuss any important strengths and limitations of the evidence including the benefits of access to IPD and any limitations arising from IPD that were not available.	22-23
Conclusions	26	Provide a general interpretation of the findings in the context of other evidence.	23-24
Implications	A4	Consider relevance to key groups (such as policy makers, service providers and service users). Consider implications for future research.	20-24
**Funding**			
Funding	27	Describe sources of funding and other support (such as supply of IPD), and the role in the systematic review of those providing such support.	25

© Reproduced with permission of the PRISMA IPD Group, which encourages sharing and reuse for non-commercial purposes

Box S1. Search strategy

Search strategy

All prospective *P. vivax* antimalarial clinical trials published between Jan 1, 2000 and June 8, 2023 were identified by the application of the key terms (listed below) through Medline (Pubmed), Web of Science, Embase and Cochrane Central. Abstracts of all references containing any mention of antimalarial drugs were manually checked to confirm prospective clinical trials, with review of full text when needed. Studies were included if they had active follow up of 28 days or more, included a treatment arm with daily primaquine given over multiple days where primaquine was commenced within 3 days of schizontocidal treatment and was given alone or coadministered with chloroquine or one of four artemisinin-based combination therapies (artemether-lumefantrine, artesunate-mefloquine, artesunate-amodiaquine, dihydroartemisinin-piperaquine) and recorded haemoglobin or haematocrit concentrations on day 0. Studies on prevention, prophylaxis, reviews, animal studies, patients with severe malaria, where schizontocidal treatment was unsupervised or where data were extracted retrospectively from medical records outside of a planned trial were excluded. The original review process that this review was based off is documented in more detail in Commons *et al*, Int J Parasitol Drug Drug Res 2017.[11] The year of the study was taken as the year in which the paper was published, although the start and end date of patient enrolment were also recorded. A *post hoc* systematic review for eligible studies in Scopus did not identify any additional eligible studies.

Key terms

Literature search (conducted June 2023) with the following key terms (version undertaken in Pubmed): vivax AND (artefenomel OR arterolane OR amodiaquine OR atovaquone OR artemisinin OR arteether OR artesunate OR artemether OR artemotil OR azithromycin OR artekin OR chloroquine OR chlorproguanil OR cycloguanil OR clindamycin OR coartem OR dapsone OR dihydroartemisinin OR duo-cotecxin OR doxycycline OR halofantrine OR lumefantrine OR lariam OR malarone OR mefloquine OR naphthoquine OR naphthoquinone OR piperaquine OR primaquine OR proguanil OR pyrimethamine OR pyronaridine OR proguanil OR quinidine OR quinine OR riamet OR sulphadoxine OR tetracycline OR tafenoquine).

Text S1. Methodology supplement

Procedures

Quantitative G6PD activity was determined as a percentage of the adjusted (healthy) male median (AMM) G6PD activity of the location for each study site. If these data were not available, thresholds were determined from the adult males with vivax malaria in the study. The timing of G6PD activity measurement was recorded as during a malaria infection or during convalescence.

Statistical analysis

Population level profile of haemoglobin over time: The population level profile of haemoglobin over time (days 0-42) in patients treated with and without primaquine was estimated using a linear mixed-effects model with non-linear terms for time derived by unadjusted fractional polynomial regression of haemoglobin on day of observation, fixed effects for age category (≤15 years and >15 years), sex, ($\log_{10}$) day 0 parasitaemia and primaquine treatment, random intercepts for study site and individual patients and random slope for time. Interaction terms between primaquine treatment and time were included to capture the differences in haemoglobin profiles over time by treatment. The covariates included in the model were identified using a directed acyclic graph (appendix p9). A joint normal distribution was assumed for the random-effects terms. Model assumptions of normality and homoskedasticity of the residuals were assessed visually. The robustness of the assumed exchangeable variance-covariance structure of the mixed-effects model was assessed by re-fitting the model with an unstructured covariance structure, which did not result in a clinically relevant change in the estimated relationship between the outcome and primaquine dose.

For the linear mixed effects model and generalised estimating equation Poisson models the assumption of a linear relationship between the continuous covariates (age and $\log_{10}$ day 0 parasitaemia) and the outcome was confirmed using univariate fractional polynomial models and the covariate was included as a categorical variable in case of a non-linear relationship.

Linear mixed-effects model of association of G6PD activity and haemoglobin at day 2-3 and day 5-7: To model the non-linear relationships between haemoglobin and G6PD activity, and haemoglobin and age and day 0 parasitaemia, a multiple fractional polynomial model was fitted (alpha=0.05, 4 degrees of freedom considered), adjusting for age, sex, $\log_{10}$ of day 0 parasitaemia, and haemoglobin at day 0, with random effects for study site. An interaction term between daily dose of primaquine and the fractional polynomial terms for G6PD activity was included in the model.

Risk of bias

The Cochrane Risk of Bias 2 tool (Higgins JPT, Savović J, Page MJ, Elbers RG, Sterne JAC. Assessing risk of bias in a randomized trial. In: Higgins JPT, Thomas J, Chandler J, et al., eds. Cochrane Handbook for Systematic Reviews of Interventions version 63 (updated February 2022): Cochrane; 2022) and the Joanna Briggs Institute Case Series tool (Munn Z, Moola S, Lisy K, Riitano D, Tufanaru C. Methodological guidance for systematic reviews of observational epidemiological studies reporting prevalence and cumulative incidence data. Int J Evid Based Healthc 2015; 13(3): 147-53) were used to assess within study risk of bias for randomised controlled trials and for single arm studies respectively. To assess inclusion bias, baseline characteristics were compared between included studies and eligible but unavailable studies. The coefficient of variation for estimates of the effect of primaquine dose on the maximum absolute change in haemoglobin between day 0 and i) day 2-3 and ii) days 5-7 were calculated after exclusion of one study at a time to assess heterogeneity of included studies.

Figure S1. Directed acyclic graph for the relationship between primaquine treatment (exposure) and haemoglobin

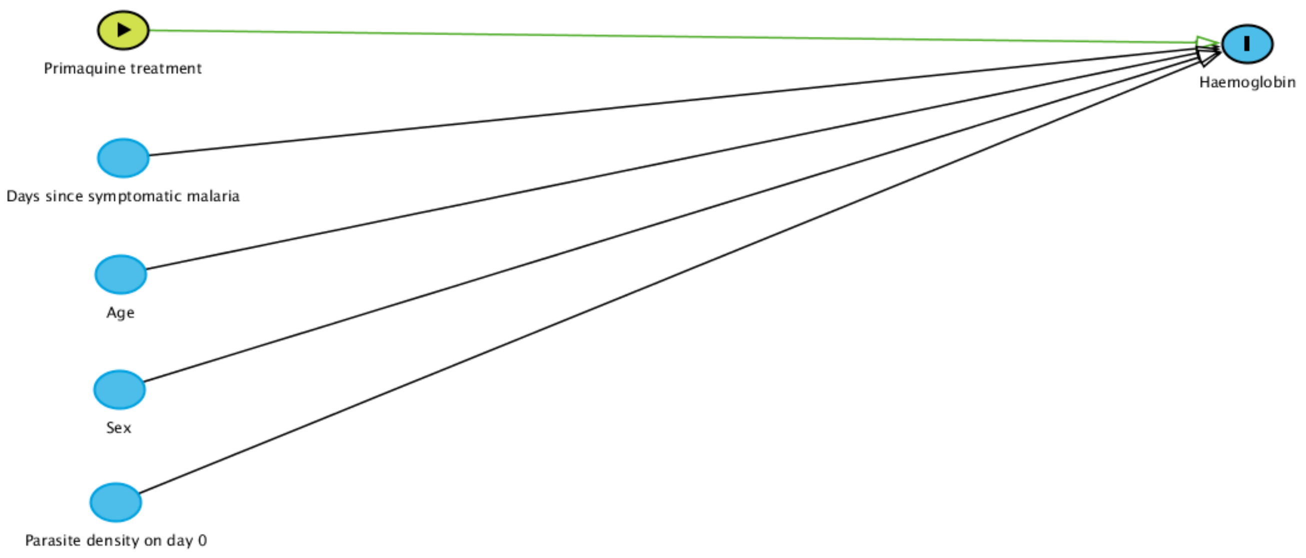

The relationship between treatment with primaquine as a binary exposure variable (green with triangle) and haemoglobin levels in g/dL (outcome, blue with I) is shown along with variables prognostic of the outcome (blue plain).

Table S1. Studies included in analysis

Author-year	Country	Recruitment Period	Age range	Follow up (days)	Treatment arms							
					Schizontocidal treatment	PQ duration	PQ total dose (mg/kg)	PQ start day	PQ supervision	Randomised	Available for haematology analysis (G6PD≥30% & baseline Hb available)	Available for quantitative G6PD analysis
Hasugian-2007[18]	Indonesia	2005	1-56	84	AsAq	14 days	4.2	2	No	Yes	51	-
					Dp	14 days	4.2	2	No	Yes	37	-
Pukrittayakamee-2010[19]	Thailand	1996-1998	14-61	28	-	7 days	3.5	0	Full	Yes	43	-
					-	7 days	7	0	Full	Yes	42	-
Barber-2013[20]	Malaysia	2010 – 2015	13-62	42	Cq/ACT	Varied	Varied	Varied	No	No	17	-
Llanos-Cuentas-2014[21]	Multinational	2010 – 2013	16-72	180	Cq	-	-	-	-	Yes	53	53
					Cq	14 days	3.5	1	Partial	Yes	50	50
Nelwan-2015[22]	Indonesia	2013	23-49	365	Dp	14 days	7	0	Full	Yes	56	-
Ley-2016[23]	Bangladesh	2014 – 2015	1-66	30	Cq	14 days	3.5	2	No	No	54	54
Saravu-2016[24]	India	2012-2015	17-75	28	Cq	14 days	3.5	0	No	No	145	-
Abreha-2017[25]	Ethiopia	2012 – 2014	1-67	365	Al	-	-	-	-	Yes	90	-
					Al	14 days	3.5	2	Partial	Yes	86	-
					Cq	-	-	-	-	Yes	100	-
					Cq	14 days	3.5	2	Partial	Yes	101	-
Awab-2017[26]	Afghanistan	2009 – 2013	2-84	390	Cq	-	-	-	-	Yes	267	-
					Cq	14 days	3.5	0	Partial	Yes	273	-
Chu-2018[27]	Thailand	2010 – 2011	1-63	365	Cq	-	-	-	-	Yes	194	-
					Cq	14 days	7	0	Full	Yes	191	-
Grigg-2018[28]	Malaysia	2013 – 2015	8m-65	230	Cq/ACT	Varied	Varied	Varied	No	No	6	-
Chu-2019[29]	Thailand	2012 – 2014	1-63	365	Cq	14 days	7	0	Full	Yes	164	14
					Cq	7 days	7	0	Full	Yes	166	10
					Dp	14 days	7	0	Full	Yes	163	7
					Dp	7 days	7	0	Full	Yes	161	11
Lacerda-2019[30]	Multinational	2013 – 2017	15-71	180	Cq	-	-	-	-	Yes	132	132
					Cq	14 days	3.5	1	Partial	Yes	129	129
Ladeia-Andrade-2019[31]	Brazil	2014 – 2015	7-60	180	Cq	7 days	3.5	0	Full	Yes	92	-
Llanos-Cuentas-2019[32]	Multinational	2014 – 2017	15-74	180	Cq	14 days	3.5	1	Partial	Yes	85	85
Rijal-2019[33]	Nepal	2015 – 2016	5-75	365	Cq	-	-	-	-	Yes	99	-
					Cq	14 days	3.5	0	Partial	Yes	105	-
Taylor-2019[14]	Multinational	2014 – 2017	9m-94	365	Cq/ACT	-	-	-	-	Yes	256	50
					Cq/ACT	14 days	7	0	Full	Yes	537	96
					Cq/ACT	7 days	7	0	Full	Yes	526	95
					Dp	-	-	-	-	Yes	196	174
					Dp	14 days	7	0	Full	Yes	382	315
					Dp	7 days	7	0	Full	Yes	387	334
Karunajeewa-unpublished	Vanuatu	2013	2-35	84	Al	-	-	-	-	Yes	9	-
					Al	14 days	3.5	0	Full	Yes	17	-

ACT – artemisinin-based combination treatment; As – artesunate; Al – artemether-lumefantrine; Aq – amodiaquine; Cq – chloroquine; Dp – dihydroartemisinin-piperaquine; Mf – mefloquine; PQ – primaquine; Primaquine supervision was categorised based on study protocols as unsupervised (no or one dose directly observed), partially supervised (≥2 doses observed) and fully supervised (all doses observed).

Table S2. Study testing and inclusion criteria

Author-year	Hb exclusion criteria	G6PD testing undertaken	G6PD activity included	G6PD data available	Timing of G6PD testing	Method of Hb measurement	Days Hb/Hct measured and data available*
Hasugian-2007[18]	Hct <15%	FST	≥30% activity	Yes	Acute	Hb	0, 7, 14
Pukrittayakamee-2010[19]	No exclusions	FST	≥30% activity	Yes	Acute	Hct	0, 1, 2, 3, 4, 5, 6, 7, 14
Barber-2013[20]	No exclusions	FST	≥30% activity	Yes	Acute	Hb	0, 1, 2, 3
Llanos-Cuentas-2014[21]	Hb <7 g/dL	Spectrophotometry	≥70% activity	Yes	Acute	Hb	0, 2, 3, 4, 5, 6, 7, 8, 9, 10, 11, 12, 13, 14
Nelwan-2015[22]	No exclusions	FST	≥30% activity	Yes	Acute	Hb	0, 3, 7, 14
Ley-2016[23]	Hb <8 g/dL	Spectrophotometry	All activities (if deficient not given PQ)	Yes	Acute	Hb	0, 2, 6, 8, 9, 10, 12
Saravu-2016[24]	No exclusions	Spectrophotometry	All activities (if deficient given weekly PQ)	Yes	Acute	Hb	0
Abreha-2017[25]	Hb <8 g/dL	FST	≥30% activity	Yes	Acute	Hb	0, 3, 7, 14
Awab-2017[26]	Hb <8 g/dL	FST	≥30% activity	Yes	Acute	Hb	0, 1, 2, 7, 14
Chu-2018[27]	Hct <25%	FST	All activities (if deficient not given PQ)	Yes	Acute	Hct	0, 14
Grigg-2018[28]	Hb <5 g/dL	FST	All activities (if deficient not given PQ)	Yes	Acute	Hb	0, 1, 2, 3, 7, 14
Chu-2019[29]†	Hct <25%	FST/ Spectrophotometry	≥30% activity	Yes	Steady state	Hct	0, 1, 2, 3, 4, 5, 6, 7, 8, 9, 12, 13, 14
Lacerda-2019[30]	Hb <7 g/dL	Spectrophotometry	≥70% activity	Yes	Acute	Hb	0, 1, 2, 3, 4, 5, 6, 7, 8, 9, 10, 11, 12, 13, 14
Ladeia-Andrade-2019[31]	Hb <8 g/dL	BinaxNow RDT	≥30% activity	Yes	Acute	Hb	0, 1, 2, 3, 7, 14
Llanos-Cuentas-2019[32]	Hb <7 g/dL	Spectrophotometry	≥70% activity	Yes	Acute	Hb	0, 2, 3, 4, 5, 6, 7, 8, 9, 10, 11, 12, 13, 14
Rijal-2019[33]	Not stated	Carestart RDT	≥30% activity	Yes	Acute	Hct	0, 1, 3, 7
Taylor-2019[14]	Hb <9 g/dL	FST/ Spectrophotometry	≥30% activity	Yes	Acute	Hb	0, 3, 4, 5, 7, 8, 9, 10, 13, 14
Karunajeewa-unpublished	Hb <5 g/dL	Carestart or BinaxNow RDT	≥30% activity	Yes	Acute	Hb	0, 1, 2, 3, 7, 10, 14

FST – fluorescent spot test; G6PD – glucose-6-phosphate dehydrogenase deficiency; Hb – haemoglobin; Hct – haematocrit; RDT – rapid diagnostic test; * For minimum 10 patients; † Spectrophotometry was only undertaken on some patients enrolled in a nested cohort study.[41]

Table S3. Study sites included in analysis

Paper	Study site	Country	Region	Lat	Long	Year Start	Year End	AMM (U/gHb)	MAP Incidence rate (per 1000 persons)	Transmission intensity*	Relapse periodicity†
Hasugian-2007[18]	Timika	Indonesia	Asia-Pacific	-4.61	136.85	2005	2005		22.61	High	High
Pukrittayakamee-2010[19]	Bangkok	Thailand	Asia-Pacific	13.76	100.50	1996	1998		2.15	Moderate‡	High
Barber-2013[20]	Sabah	Malaysia	Asia-Pacific	5.98	116.08	2011	2011		0.21	Low	High
Llanos-Cuentas-2014[21]	Mae Sot	Thailand	Asia-Pacific	16.72	98.58	2011	2013	8.44	3.07	Moderate	High
Llanos-Cuentas-2014[21]	Bangkok	Thailand	Asia-Pacific	13.76	100.50	2011	2013	13.09	0.16	Low	High
Llanos-Cuentas-2014[21]	Lucknow	India	Asia-Pacific	26.85	80.95	2011	2013	8.74	2.84	Moderate	Low
Llanos-Cuentas-2014[21]	Chennai	India	Asia-Pacific	13.06	80.25	2011	2013	8.74	1.46	Moderate	Low
Llanos-Cuentas-2014[21]	Manaus	Brazil	Americas	-3.12	-60.02	2011	2013	6.82	42.81	High	Low
Llanos-Cuentas-2014[21]	Bikaner	India	Asia-Pacific	28.02	73.31	2011	2013	8.74	2.85	Moderate	Low
Llanos-Cuentas-2014[21]	Iquitos	Peru	Americas	-3.74	-73.25	2011	2013	8.65	40.49	High	Low
Nelwan-2015[22]	Sragen	Indonesia	Asia-Pacific	-7.42	111.02	2013	2013		0.07	Low	High
Ley-2016[23]	Alikadam Upazilla	Bangladesh	Asia-Pacific	23.70	90.44	2014	2015	7.03	0.59	Low	Low
Saravu-2016[24]	Udupi taluk	India	Asia-Pacific	13.48	74.71	2012	2015		2.16	Moderate	Low
Abreha-2017[25]	Batu	Ethiopia	Africa	6.67	39.42	2013	2013		85.49	High	Low
Abreha-2017[25]	Bishoftu	Ethiopia	Africa	8.73	39.01	2013	2013		85.49	High	Low
Awab-2017[26]	Jalalabad	Afghanistan	Asia-Pacific	34.43	70.46	2009	2014		40.12	High	Low
Chu-2018[27]	Mae Sot	Thailand	Asia-Pacific	16.72	98.58	2010	2010		2.15	Moderate	High
Grigg-2018[28]	Kudat	Malaysia	Asia-Pacific	6.89	116.85	2014	2014		0.11	Low	High
Chu-2019[29]	Mae Sot	Thailand	Asia-Pacific	16.72	98.58	2014	2014	7.03	3.09	Moderate	High
Lacerda-2019[30]	Tak	Thailand	Asia-Pacific	16.88	99.13	2013	2016	8.03	0.63	Low	High
Lacerda-2019[30]	Rio Tuba	Philippines	Asia-Pacific	8.54	117.44	2013	2016	7.72	2.26	Moderate	High
Lacerda-2019[30]	Porto Velho	Brazil	Americas	-8.76	-63.90	2013	2016	7.87	9.23	Moderate	Low
Lacerda-2019[30]	Oddar Meanchey Province	Cambodia	Asia-Pacific	14.16	103.82	2013	2016	7.88	7.38	Moderate	High
Lacerda-2019[30]	Iquitos	Peru	Americas	-3.74	-73.25	2013	2016	8.92	89.80	High	Low
Lacerda-2019[30]	Manaus	Brazil	Americas	-3.12	-60.02	2013	2016	7.87	41.14	High	Low
Lacerda-2019[30]	Jimma	Ethiopia	Africa	7.67	36.84	2013	2016	9.01	40.53	High	Low
Lacerda-2019[30]	Gondar	Ethiopia	Africa	12.60	37.45	2013	2016	9.01	6.72	Moderate	Low
Ladeia-Andrade-2019[31]	Mancio Lima	Brazil	Americas	-7.61	-72.91	2014	2014		47.40	High	Low
Llanos-Cuentas-2019[32]	Umphang	Thailand	Asia-Pacific	15.88	98.92	2015	2016	7.98	1.08	Moderate	High
Llanos-Cuentas-2019[32]	Monteria	Colombia	Americas	8.75	-75.88	2015	2016	8.56	5.36	Moderate	Low
Llanos-Cuentas-2019[32]	Ho Chi Minh City	Vietnam	Asia-Pacific	10.82	106.63	2015	2016	7.62	0.01	Low§	High
Llanos-Cuentas-2019[32]	Thailand4	Thailand	Asia-Pacific			2015	2016	7.98	0.13	Low	High
Llanos-Cuentas-2019[32]	Cali	Colombia	Americas	3.45	-76.53	2015	2016	8.56	1.93	Moderate	Low
Llanos-Cuentas-2019[32]	Manaus	Brazil	Americas	-3.12	-60.02	2015	2016	7.69	18.55	High	Low
Llanos-Cuentas-2019[32]	Iquitos	Peru	Americas	-3.74	-73.25	2015	2016	8.09	58.05	High	Low
Rijal-2019[33]	Jhapa	Nepal	Asia-Pacific	26.55	87.89	2016	2016		0.12	Low	High
Rijal-2019[33]	Kailali	Nepal	Asia-Pacific	28.83	80.90	2016	2016		0.22	Low	High
Taylor-2019[14]	Krong Pa	Vietnam	Asia-Pacific	13.22	108.67	2015	2017		0.18	Low	High
Taylor-2019[14]	Dak O	Vietnam	Asia-Pacific	12.00	107.50	2015	2017		0.24	Low	High

Taylor-2019[14]	Bu Gia Map	Vietnam	Asia-Pacific	12.04	107.05	2015	2017		0.24	Low	High
Taylor-2019[14]	Hanura	Indonesia	Asia-Pacific	-5.53	105.24	2015	2017	7.51	1.01	Moderate	High
Taylor-2019[14]	Tanjung Leidong	Indonesia	Asia-Pacific	2.77	99.98	2015	2017	8.44	1.03	Moderate	High
Taylor-2019[14]	Arba Minch	Ethiopia	Africa	6.01	37.54	2015	2017	12.12	17.80	High	Low
Taylor-2019[14]	Laghman	Afghanistan	Asia-Pacific	34.70	70.15	2015	2017		97.70	High	Low
Taylor-2019[14]	Metahara	Ethiopia	Africa	8.90	39.92	2015	2017		26.02	High	Low
Taylor-2019[14]	Jalalabad	Afghanistan	Asia-Pacific	34.43	70.46	2015	2017		116.49	High	Low
Karunajeewa-unpublished	Nambauk	Vanuatu	Asia-Pacific	-15.45	167.08	2013	2013		24.58	High	High
Karunajeewa-unpublished	Port Olry	Vanuatu	Asia-Pacific	-15.04	167.07	2013	2013		24.58	High	High
Karunajeewa-unpublished	Luganville	Vanuatu	Asia-Pacific	-15.51	167.20	2013	2013		24.58	High	High

AMM – adjusted male median; Lat – latitude; Long – longitude; MAP – malaria Atlas Project; The adjusted male median G6PD activity was derived from the median of male G6PD activity measurements for sites with at least 36 observations. If there were <36 observations per site, the AMM was derived from observations in the same country and study. *Transmission intensity is classified as low (an incidence rate of <1 per 1000 persons), moderate (1 to <10 per 1000 persons), high (≥10 per 1000 persons); † Short relapse periodicity ≤47 days; ‡Study done in Bangkok but the majority of patients acquired malaria from the Western border of Thailand where there was high transmission; §Study site in Ho Chi Minh City where there is minimal to no transmission but patients presumed to be from provinces.

Table S4. Reasons for studies not being included in analysis

Reason	Number of studies	Studies[*]
Data not available by August 23, 2021	8	[49-56]
Investigators unable to be contacted	2	[57,58]
Missing minimum data for inclusion	1	[59]
Initial investigator response but no data provided	1	[60]
No response from investigators	4	[61-64]
Data available but excluded on patient-level factors	2	[65,66]

***** References of studies not included are provided in References S1

Table S5. Studies eligible for the analysis but not included

First Author	Treatment Arms	Number of Sites	Region	Country	Follow up (days)	Randomised	Recruitment period	Treatment arms*	Pv patients enrolled	Treated with PQ	Female (%)	Mean Age (SD)	Median Age (range)	Reasons for exclusion
Pukrittayakamee-2000[49]	9	1	Asia-Pacific	Thailand	28	Yes	1992-1998	Arm; As; Hal; Mf; Qu; SP; Cq; Pq_3.5_14d_D0; Cq_Pq_3.5_14d_D3	207	60	0	25 (9)		Data not available
Mohapatra-2002[57]	1	1	Asia-Pacific	India	365	No	1998-2000	Cq_Pq_3.5_14d_DX	110	110	36.4	Not stated		Unable to be contacted
Rajgor-2003[61]	2	1	Asia-Pacific	India	180	Yes	1998-2000	Cq; Cq_Pq_3.5_14d_D4	273	131	12.1	Not stated		No response from investigators
Walsh-2004[58]	5	1	Asia-Pacific	Thailand	168	Yes	1998-1999	Cq_Tq; Cq_Tq; Cq_Tq; Cq; Cq_Pq_3.5_14d_D2	80	12	68	Not stated		Unable to be contacted
Tasanor-2006[64]	2	1	Asia-Pacific	Thailand	28	Yes	2002-2004	Cq_Pq_3.5_14d_D0; Qu_Pq_3.5_14d_D0	58	58	29.0	Not stated		No response from investigators
Carmona-Fonseca-2010[50]	2	1	Americas	Colombia	120	Yes	2005-2008	Cq_Pq_3.5_7d_D1; Cq_Pq_3.5_3d_D1	79	79	Not stated	Not stated		Data not available
Daneshvar-2010[59]	1	1	Asia-Pacific	Malaysia	28	No	2006-2007	Cq_Pq_3.5_14d_D1	23	23	0	38.5 (7.6)		Missing minimum data
Saravu-2012[51]	1	1	Asia-Pacific	India	28	No	2007-2009	Cq_Pq_3.5_14d_D2	110	110	26.3	Not stated	28.5 (23-29)	Data not available
Ganguly-2013[62]	2	1	Asia-Pacific	India	42	Yes	2011-2012	Cq; Cq_Pq_3.5_14d_D0	250	125	10.8	25.2 (-)		No response from investigators
Macareo-2013[63]	2	1	Asia-Pacific	Thailand	90	Yes	Not stated	Cq_Pq_7.0_14d_D0; Cq_Tnd	20	6	Not stated	Not stated		No response from investigators
Negreiros-2016[60]	1	1	Americas	Brazil	168	No	2014	Cq_Pq_3.5_7d_D0	119	119	45.4	Not stated	23.4 (5-67.3)	No data provided
Dharmawardena-2017[52]	1	1	Asia-Pacific	Sri Lanka	365	No	2015-2016	Cq_Pq_3.5_14d_D2	32	32	6.8		35.5 (13-66)	Data not available
Poespoprodjo-2022[53]	2	1	Asia-Pacific	Indonesia	180	Yes	2016-2018	Dp_ Pq(sup)_7.0_14d_D0; Dp_ Pq(unsup)_7.0_14d_D0	419	419	47	Not stated	17.2 (7.4-33.1)	Data not available by August 23, 2021
Mekonnen-2023[54]	1	1	Africa	Ethiopia	42	No	2019-2020	Cq_Pq_3.5_14d_D0	102	102	50	Not stated	13.5	Data not available by August 23, 2021
Moore-2023[55]	3	1	Asia-Pacific	PNG	63	Yes	2013-2018	Al_Pq_7.0_14d_D0; Al_Pq_7.0_7d_D0; Al_Pq_7.0_3.5d_D0	73	73	40	Not stated	6.6	Data not available by August 23, 2021
Sutanto-2023[56]	3	2	Asia-Pacific	Indonesia	180	Yes	2018-2019	Dp; Dp_Pq_3.5_14d_D1; Dp_Tq	150	50	0	28.6 (5.6)		Data not available by August 23, 2021

Al – artemether lumefantrine; Anq – artemisinin-naphthoquine; Arm – artemether; Artm – arterolane maleate; As – artesunate; Bq – bulaquine; Cq – chloroquine; Dp – dihydroartemisinin-piperaquine; Hal – halofantrine; Mf – mefloquine; Pip – piperaquine; PNG – Papua New Guinea; PQ/Pq – primaquine; Pv – *P. vivax*; Qu – Quinine; SD – standard deviation; SP – sulfadoxine-pyrimethamine; sup – supervised; Tnd – tinidazole; Tq – tafenoquine; unsup – unsupervised; *Treatment code describes (schizontocidal drug)_(hypnozoitocidal drug)_(total primaquine dose)_(duration of primaquine treatment eg 14d = 14 days)_(primaquine start day)

Figure S2. Location of study sites

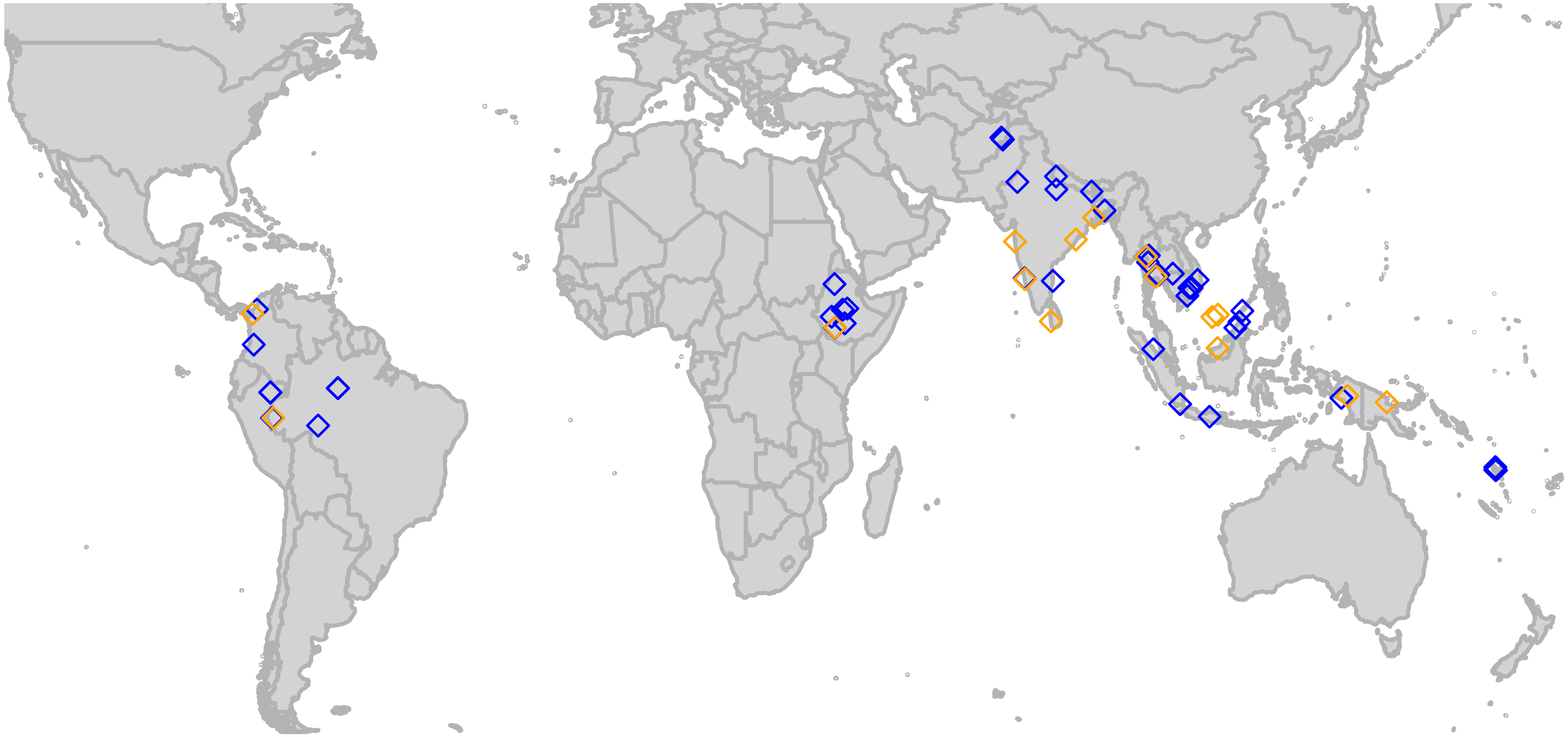

Blue – included; orange – eligible but not included

Table S6. Comparison of baseline characteristics between included and eligible but not included studies

Characteristic	Included studies (n=18)	Eligible but not included studies* (n=16)
Region#		
Asia-Pacific, studies (%)	14.5 (81%)	13 (81%)
Africa, studies (%)	1.5 (8%)	0 (0%)
The Americas, studies (%)	2 (11%)	3 (19%)
Year of enrolment†		
Pre-2009, studies (%)	2 (11%)	7 (47%)‡
2009-2017, studies (%)	16 (89%)	8 (53%)‡
Follow up duration (days)		
≤42	4 (22%)	6 (38%)
>42 to <180	2 (11%)	5 (31%)
180	4 (22%)	3 (19%)
>180	8 (44%)	2 (13%)
Age (years), median (IQR)	19 (12-30)	23 (17-25)§
Female, % of patients, median (IQR)	36¶	29 (11-47) ||

IQR – interquartile range; # Multinational studies are considered as a proportion of the number of study sites within each region; * Age, and female percentage of targeted studies calculated using frequency weighted mean or median according to number of patients; † Year of enrolment defined as the year study enrolment completed; ‡ Year of enrolment not available for categorisation from 1 study; § Mean or median age not available from 6 studies; ¶ No IQR presented as data based on actual percentage of female patients in included studies; || Percentage not available from 2 studies.

Table S7. Risk of bias assessment in 14 included randomised controlled studies

Author-year	Bias from randomisation	Bias due to deviation from intervention	Bias from missing outcome		Bias in measurement of the outcome	Bias in selection of the reported results	Overall bias	Follow up to 180 days	Comparison of no PQ to PQ
Hasugian-2007[18]									
Pukrittayakamee-2010[19]									
Llanos-Cuentas-2014[21]									
Nelwan-2015[22]									
Abreha-2017[25]									
Awab-2017[26]									
Chu-2018[27]									
Chu-2019[29]		*							
Lacerda-2019[30]									
Ladeia-Andrade-2019[31]									
Llanos-Cuentas-2019[32]									
Rijal-2019[33]		*							
Taylor-2019[14]									
Karunajeewa-unpublished									

Green – low risk of bias; Red – high risk of bias; Orange – unclear risk of bias; Grey – not applicable; Assessed according to the Cochrane Risk of Bias 2 tool for randomised controlled trials (Higgins JPT, Savović J, Page MJ, Elbers RG, Sterne JAC. Assessing risk of bias in a randomized trial. In: Higgins JPT, Thomas J, Chandler J, et al., eds. Cochrane Handbook for Systematic Reviews of Interventions version 63 (updated February 2022): Cochrane; 2022); * Studies analysed per protocol but all data available for these meta-analyses; PQ – primaquine.

Table S8. Risk of bias assessment in 4 included single arm observational studies

Author-year	Clear criteria for inclusion	Condition measured in reliable way	Valid methods for condition	Consecutive inclusion	Complete inclusion	Demographics reported	Clinical information reported	Outcomes reported	Site description	Analysis appropriate	Follow up to 180 days	Comparison of no PQ to PQ
Barber-2013[20]	Green	Green	Green	Green	Green	Green	Green	Green	Green	Green	Red	Grey
Ley-2016[23]	Green	Green	Green	Green	Green	Green	Green	Green	Green	Green	Red	Grey
Saravu-2016[24]	Green	Green	Green	Green	Orange	Green	Green	Green	Green	Green	Red	Grey
Grigg-2018[28]	Orange	Green	Green	Orange	Orange	Red	Red	Green	Green	Green	Red	Grey

Green – yes (low risk of bias); Red – no (higher risk of bias); Orange – unclear; Grey – not applicable; Assessed according to the Joanna Briggs Institute Case Series tool for single arm studies (Munn Z, Moola S, Lisy K, Riitano D, Tufanaru C. Methodological guidance for systematic reviews of observational epidemiological studies reporting prevalence and cumulative incidence data. Int J Evid Based Healthc 2015; 13(3): 147-53); The appropriateness of analysis was considered appropriate for all studies given that the individual patient data were re-analysed as part of these meta-analyses; PQ – primaquine.

Table S9. Patients experiencing primary or secondary haematological outcomes or severe anaemia

ID	Sex	Age (years)	PQ daily dose category	G6PD activity (%)	Timing of G6PD activity measurement	G6PD genotype	Hb day 0 (g/dL)	Lowest recorded Hb days 1-14 (g/dL)	Day of lowest recorded Hb	Maximum absolute fall in Hb from day 0 to days 1-14 (g/dL)	Hb day 2-3 (g/dL)	Hb days 5-7 (g/dL)	Outcome			
													>25% fall to <7 g/dL	>5 g/dL fall	Fall to <5 g/dL	Blood transfusion
1	Female	13	High	61.53	Steady state	Het	8.8	4.4	5	4.4	5.5	4.4	Yes	No	Yes	Yes
2	Male	35	High	≥30%	Acute		5.5	4.8	1	0.7	5.1	5.9	No	No	Yes	No
3	Male	5	Intermediate	≥30%	Acute		10.1	5.9	3	4.2	5.9	7.8	Yes	No	No	No
4	Male	15	Intermediate	≥30%	Acute		9.4	5.9	2	3.5	5.9	.	Yes	No	No	No
5	Male	57	High	≥30%	Acute		11.6	5.9	2	5.7	5.9	10.5	Yes	Yes	No	No
6	Female	30	Intermediate	≥30%	Acute		12	5.9	7	6.1	9.8	5.9	Yes	Yes	No	No
7	Female	5	No PQ	≥30%	Acute		10.3	6.3	3	4.0	6.3	9.9	Yes	No	No	.
8	Female	12	High	≥30%	Acute		10.5	6.3	7	4.2	9.0	6.3	Yes	No	No	No
9	Female	21	High	94.78	Steady state	Het	9.8	6.3	5	3.5	7.1	6.3	Yes	No	No	Yes
10	Female	10	Intermediate	≥30%	Acute		9.0	6.7	5	2.3	7.1	6.7	Yes	No	No	No
11	Female	13	High	≥30%	Acute		11.0	6.7	6	4.3	7.8	6.7	Yes	No	No	No
12	Male	3	Intermediate	≥30%	Acute		10.2	6.8	3	3.4	6.8	7.7	Yes	No	No	No
13	Female	10	High	37.41	Acute		11.6	6.9	3	4.7	6.9	8.4	Yes	No	No	No
14	Male	28	Intermediate	≥30%	Acute		12.9	7.1	4	5.8	14.0	14.8	No	Yes	No	No
15	Female	10	Intermediate	75.86	Steady state	Het	12.6	7.5	5	5.1	10.1	7.5	No	Yes	No	No
16	Male	15	High	≥30%	Acute		14.8	8.6	6	6.2	12.8	8.6	No	Yes	No	No
17	Male	21	Intermediate	≥30%	Acute		14.6	8.6	4	6.0	11.7	12.5	No	Yes	No	No
18	Female	12	High	130.48	Acute		13.9	8.8	7	5.1	11.4	8.8	No	Yes	No	No
19	Male	27	Intermediate	≥30%	Acute		15.1	9.4	5	5.7	11.7	9.4	No	Yes	No	No
20	Male	21	No PQ	≥30%	Acute		16.0	10.0	2	6.0	10.0	16.0	No	Yes	No	No
21	Male	27	Intermediate	≥30%	Acute		17.8	10.9	1	6.9	17.1	16.3	No	Yes	No	No
22	Male	67	High	≥30%	Acute		16.6	11.0	10	5.6	12.8	12.9	No	Yes	No	No
23	Male	52	Intermediate	≥30%	Acute		16.8	11.3	3	5.5	11.3	14.4	No	Yes	No	No
24	Male	45	High	≥30%	Acute		17.0	11.9	13	5.1	13.8	13.2	No	Yes	No	No
25	Male	18	Intermediate	≥30%	Acute		17.5	12.2	2	5.3	12.2	12.8	No	Yes	No	No

G6PD – glucose-6-phosphate dehydrogenase; Hb – haemoglobin; Het – heterozygous for G6PD deficiency; PQ – primaquine; WT – wild type for G6PD. Hb converted from haematocrit if haemoglobin not recorded.

Figure S3. Haemoglobin profiles of patients with ≥30% G6PD activity treated with or without primaquine

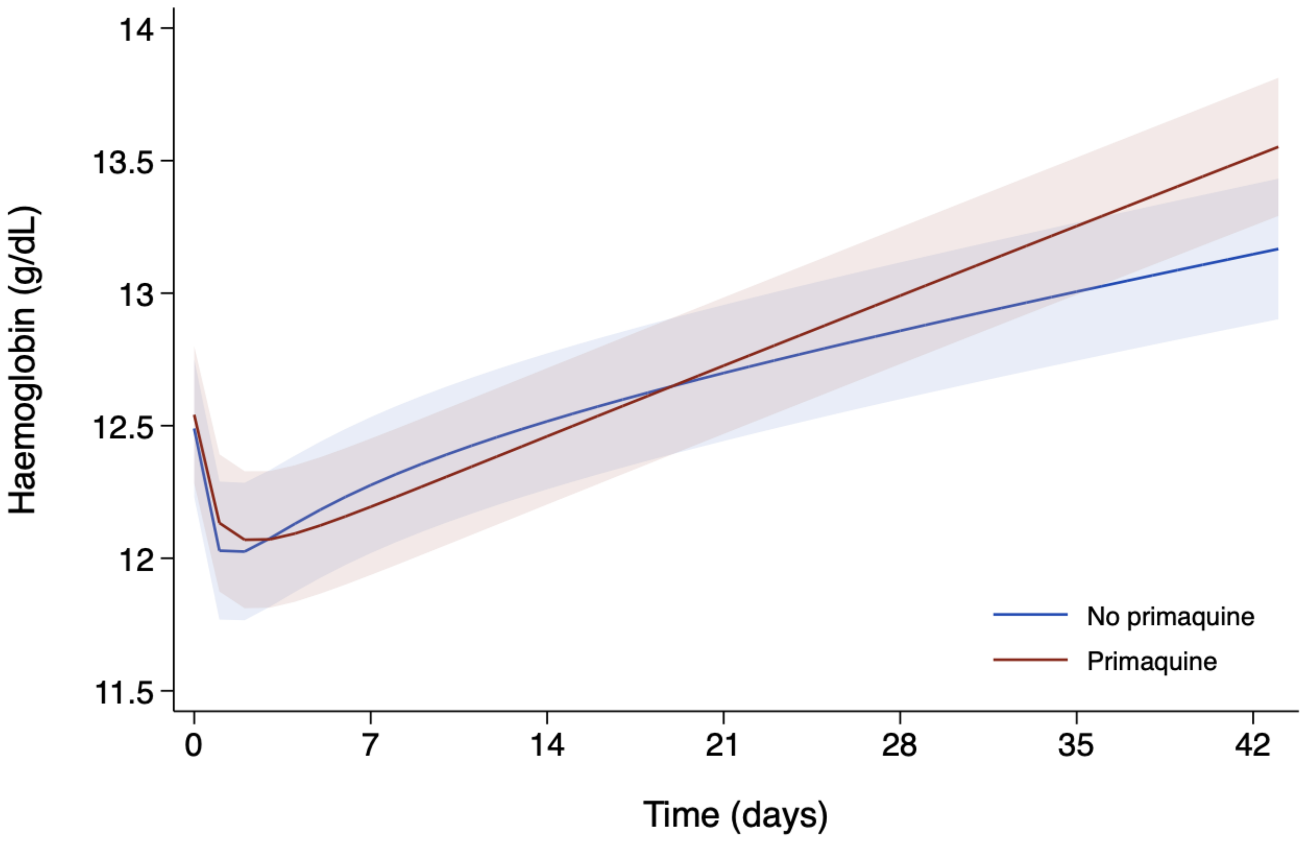

Profiles were generated from a linear mixed effects model with fractional polynomial terms for the relationship between haemoglobin and time and interaction terms for primaquine treatment and time, controlling for age, sex and baseline parasitaemia, with random effects for study site, individual patients and the fractional polynomial terms. Models were fit to data from 1,419 patients treated without primaquine and 3,368 patients treated with primaquine on day 0.

Tables S10. Sensitivity analyses of change in haemoglobin from day 0 to day 2-3 and day 0 to days 5-7

	Excluding one study site at a time		Restricted to patients where actual primaquine daily dose administered was known		
	**Estimated range of mean Hb change from day 0 (g/dL)**	**Coefficient of variation (%)**	**Adjusted estimated mean change in Hb (g/dL)**	**(95% CI)**	**Sample size**
Hb change from day 0 to day 2-3					4191
Primaquine daily dose					
No PQ	-0.6 to -0.6	-1.9	-0.6	(-0.7, -0.5)	
Low daily dose	-0.7 to -0.6	-2.1	-0.6	(-0.8, -0.5)	
Intermediate daily dose	-0.6 to -0.5	-2.5	-0.5	(-0.7, -0.4)	
High daily dose	-0.6 to -0.5	-3.5	-0.5	(-0.6, -0.3)	
Hb change from day 0 to days 5-7					4111
Primaquine daily dose					
No PQ	-0.3 to -0.2	-4.6	-0.2	(-0.4, -0.1)	
Low daily dose	-0.3 to -0.2	-4.2	-0.2	(-0.4, -0.1)	
Intermediate daily dose	-0.2 to -0.2	-6.9	-0.1	(-0.3, 0.0)	
High daily dose	-0.3 to -0.2	-8.8	-0.1	(-0.3, 0.0)	

CI – Confidence Interval; Hb – haemoglobin; PQ - primaquine

Table S11. Risk of and adjusted risk ratios for anaemia on day 2-3 and days 5-7 in patients with G6PD ≥30%

	No PQ	Low daily dose PQ (<0.375 mg/kg/day)	Intermediate daily dose PQ (≥0.375 & <0.75 mg/kg/day)	High daily dose PQ (≥0.75 mg/kg/day)
Development of mild, moderate or severe anaemia on day 2-3 in patients, (n (%))				
Hb starts ≥11 and drops to 8-11 g/dL	88/957 (9.2%)	48/701 (6.8%)	186/1,189 (15.6%)	172/1,018 (16.9%)
Hb starts ≥11 and drops to 5-8 g/dL	1/957 (0.1%)	1/701 (0.1%)	0/1,189 (0.0%)	4/1,018 (0.4%)
Hb starts ≥11 and drops to <5 g/dL	0/957 (0.0%)	0/701 (0.0%)	0/1,189 (0.0%)	0/1,018 (0.0%)
Adjusted risk ratios for mild, moderate or severe anaemia on day 2-3				
Adjusted RR	1.0	0.8	1.2	1.2
(95% CI)		(0.7, 1.0)	(0.9, 1.7)	(0.8, 1.9)
p-value		0.11	0.27	0.41
Development of mild, moderate or severe anaemia on days 5-7, (n (%))				
Hb starts ≥11 and drops to 8-11 g/dL	54/945 (5.7%)	33/697 (4.7%)	150/1,154 (13.0%)	166/997 (16.6%)
Hb starts ≥11 and drops to 5-8 g/dL	0/945 (0.0%)	0/697 (0.0%)	3/1,154 (0.3%)	2/997 (0.2%)
Hb starts ≥11 and drops to <5 g/dL	0/945 (0.0%)	0/697 (0.0%)	0/1,154 (0.0%)	0/997 (0.0%)
Adjusted risk ratios for mild, moderate or severe anaemia on days 5-7				
Adjusted RR	1.0	0.9	1.3	1.5
(95% CI)		(0.7, 1.2)	(1.0, 1.7)	(0.9, 2.4)
p-value		0.49	0.05	0.10

Hb – Haemoglobin, RR – Risk ratio. *Adjusted risk ratios derived from generalised estimating equation Poisson models including haemoglobin on day 0, age category, sex and (log) baseline parasite density with clustering by study site, exchangeable correlation and robust standard error estimates. The models for day 2-3 was fit to data from 3,865 individuals with day 0 Hb ≥11 g/dL, and for days 5-7 was fit to data from 3,793 individuals with day 0 Hb ≥11 g/dL.

Table S12. Demographics and baseline characteristics for patients with G6PD activity ≥30% to <70%

	Overall<br>N=51	No PQ<br>N=11	Low daily dose PQ (<0.375 mg/kg/day)<br>N=3	Intermediate daily dose PQ (≥0.375 to <0.75 mg/kg/day)<br>N=15	High daily dose PQ (≥0.75 mg/kg/day)<br>N=22
Sex					
Male	23 (45%)	9 (82%)	1 (33%)	6 (40%)	7 (32%)
Female	28 (55%)	2 (18%)	2 (67%)	9 (60%)	15 (68%)
Age (years)					
Median (IQR)	19.0 (11.0-33.4)	24.3 (5.0-42.6)	17.0 (14.0-17.0)	21.0 (11.0-48.7)	17.3 (11.0-27.0)
<5	4 (8%)	3 (27%)	0 (0%)	1 (7%)	0 (0%)
5-<15	15 (29%)	0 (0%)	1 (33%)	5 (33%)	9 (41%)
≥5	32 (63%)	8 (73%)	2 (67%)	9 (60%)	13 (59%)
Enrolment variables					
G6PD activity (%)	63.4 (55.4-67.0)	63.6 (56.8-67.4)	67.0 (63.4-69.4)	63.5 (52.9-67.4)	61.3 (55.2-66.4)
Weight (kg)	44.6 (27.7-55.9)	52.4 (16.2-60.7)	44.0 (42.0-56.0)	46.0 (24.0-50.9)	42.0 (30.4-50.0)
Malnutrition*	1 (25%)	1 (33%)		0 (0%)	
Presence or recent history of fever	43 (84%)	7 (64%)	1 (33%)	13 (87%)	22 (100%)
Parasitaemia, parasites/mL	2880.0 (714.8-6880.0)	3877.8 (714.8-15418.5)	52.0 (35.0-4455.0)	3255.6 (1322.2-6880.0)	2128.0 (672.0-6785.2)
Haemoglobin, g/dL	12.6 (11.3-14.0)	12.2 (11.0-14.1)	11.5 (10.9-18.6)	12.4 (10.3-13.9)	13.0 (11.9-14.0)
Schizontocidal treatment					
Chloroquine	14 (27%)	3 (27%)	3 (100%)	3 (20%)	5 (23%)
Dihydroartemisinin-piperaquine	37 (73%)	8 (73%)	0 (0%)	12 (80%)	17 (77%)
Primaquine dosing					
PQ total mg/kg dose	7.2 (6.4-8.1)		4.8 (3.8-5.0)	7.4 (6.6-8.4)	7.3 (6.6-8.0)
PQ daily mg/kg dose	0.9 (0.5-1.1)		0.3 (0.3-0.4)	0.5 (0.5-0.6)	1.1 (1.0-1.2)
PQ dose derived from administered dose	38 (95%)		1 (33%)	15 (100%)	22 (100%)
PQ started on: Day 0	35 (88%)		0 (0.0%)	14 (93%)	21 (95%)
Day 1	1 (3%)		1 (33%)	0 (0.0%)	0 (0.0%)
Day 2	4 (10%)		2 (67%)	1 (7%)	1 (5%)
PQ duration: 14 days	18 (45%)		3 (100%)	15 (100%)	0 (0%)
7-10 days	22 (55%)		0 (0%)	0 (0%)	22 (100%)
Relapse periodicity†					
Low periodicity	4 (8%)	3 (27%)	0 (0%)	0 (0%)	1 (5%)
High periodicity	47 (92%)	8 (73%)	3 (100%)	15 (100%)	21 (95%)
Region					
Africa	1 (2%)	0 (0%)	0 (0%)	0 (0%)	1 (5%)
Asia-Pacific	50 (98%)	11 (100%)	3 (100%)	15 (100%)	21 (95%)

G6PD – glucose-6-phosphate dehydrogenase; IQR – interquartile range; PQ – primaquine; Data recorded as number (%) or median (IQR); Primaquine dosing is based on 40 patients administered primaquine; Data were not available for 47 patients on malnutrition; *The nutritional status of children aged <5 years of age was calculated as a weight-for-age z-score, using the igrowup package developed by WHO,[34] with z-scores <-2 classified as having malnutrition. Z scores were considered missing if <-6 or >6. †The relapsing phenotype of vivax, referred to as relapse periodicity, was classified by geographic region as low or high, according to a median interval from first illness to relapse above and below 47 days respectively (Battle *et al*, Malaria Journal 2014).

Table S13. Demographics and baseline characteristics for patients with G6PD activity ≥70%

	Overall<br>N=1,563	No PQ<br>N=398	Low daily dose PQ (<0.375 mg/kg/day)<br>N=294	Intermediate daily dose PQ (≥0.375 to <0.75 mg/kg/day)<br>N=445	High daily dose PQ (≥0.75 mg/kg/day)<br>N=426
Sex					
Male	916 (58.6%)	247 (62.1%)	212 (72.1%)	244 (54.8%)	213 (50.0%)
Female	647 (41.4%)	151 (37.9%)	82 (27.9%)	201 (45.2%)	213 (50.0%)
Age (years)					
Median (IQR)	19.4 (12.1-33.0)	24.0 (14.9-36.2)	32.5 (22.0-46.0)	15.1 (9.6-25.0)	15.0 (10.0-23.3)
<5	85 (5.4%)	17 (4.3%)	0 (0.0%)	35 (7.9%)	33 (7.7%)
5-<15	442 (28.3%)	83 (20.9%)	1 (0.3%)	179 (40.2%)	179 (42.0%)
≥5	1,036 (66.3%)	298 (74.9%)	293 (99.7%)	231 (51.9%)	214 (50.2%)
Enrolment variables					
G6PD activity	101.4 (91.0-114.2)	101.5 (91.3-115.7)	103.6 (94.5-111.9)	100.6 (89.0-115.6)	100.2 (90.0-114.5)
Weight (kg)	50.7 (31.5-60.9)	54.0 (41.4-63.5)	61.5 (53.0-69.0)	42.4 (23.4-54.8)	42.4 (24.0-54.8)
Malnutrition*	20 (21.5%)	3 (17.6%)		11 (26.8%)	6 (17.1%)
Presence or recent history of fever	1,351 (86.4%)	325 (81.7%)	229 (77.9%)	408 (91.7%)	389 (91.3%)
Parasitaemia, parasites/mL	4158.0 (1170.4-10666.0)	4665.3 (1611.1-11555.6)	3856.0 (960.0-9380.0)	3911.1 (877.8-11711.1)	3925.9 (1203.7-9800.0)
Haemoglobin, g/dL	12.8 (11.6-13.9)	13.0 (11.7-14.0)	13.1 (12.1-14.1)	12.6 (11.4-13.7)	12.6 (11.4-13.9)
Schizontocidal treatment					
Artemether-lumefantrine	8 (0.5%)	3 (0.8%)	0 (0.0%)	1 (0.2%)	4 (0.9%)
Chloroquine	749 (47.9%)	229 (57.5%)	288 (98.0%)	132 (29.7%)	100 (23.5%)
Dihydroartemisinin-piperaquine	806 (51.6%)	166 (41.7%)	6 (2.0%)	312 (70.1%)	322 (75.6%)
Primaquine dosing					
PQ total mg/kg dose	6.9 (4.7-7.7)		3.4 (3.1-4.0)	7.2 (6.7-8.0)	7.2 (6.7-8.2)
PQ daily mg/kg dose	0.6 (0.4-1.0)		0.3 (0.2-0.3)	0.5 (0.5-0.6)	1.0 (1.0-1.2)
PQ dose derived from administered dose	1,113 (95.5%)		265 (90.1%)	423 (95.1%)	425 (99.8%)
PQ started on: Day 0	850 (73.0%)		9 (3.1%)	418 (93.9%)	423 (99.3%)
Day 1	259 (22.2%)		255 (86.7%)	4 (0.9%)	0 (0.0%)
Day 2	56 (4.8%)		30 (10.2%)	23 (5.2%)	3 (0.7%)
PQ duration: 14 days	732 (62.8%)		294 (100.0%)	437 (98.2%)	1 (0.2%)
7-10 days	433 (37.2%)		0 (0.0%)	8 (1.8%)	425 (99.8%)
Relapse periodicity†					
Low periodicity	577 (36.9%)	190 (47.7%)	197 (67.0%)	96 (21.6%)	94 (22.1%)
High periodicity	986 (63.1%)	208 (52.3%)	97 (33.0%)	349 (78.4%)	332 (77.9%)
Region					
Africa	265 (17.0%)	64 (16.1%)	13 (4.4%)	94 (21.1%)	94 (22.1%)
Americas	299 (19.1%)	119 (29.9%)	178 (60.5%)	2 (0.4%)	0 (0.0%)
Asia-Pacific	999 (63.9%)	215 (54.0%)	103 (35.0%)	349 (78.4%)	332 (77.9%)

G6PD – glucose-6-phosphate dehydrogenase; IQR – interquartile range; PQ – primaquine; Data recorded as number (%) or median (IQR); Primaquine dosing is based on 1,165 patients administered primaquine; Data were not available for 1,470 patients on malnutrition; *The nutritional status of children aged <5 years of age was calculated as a weight-for-age z-score, using the igrowup package developed by WHO,[34] with z-scores <-2 classified as having malnutrition. Z scores were considered missing if <-6 or >6; †The relapsing phenotype of vivax, referred to as relapse periodicity, was classified by geographic region as low or high, according to a median interval from first illness to relapse above and below 47 days respectively (Battle *et al*, Malaria Journal 2014).

Figure S4. A) Model of G6PD activity compared with haemoglobin on days 5-7 in patients with quantitative G6PD activity ≥30% and B) observed G6PD activity versus haemoglobin on day 5-7 in patients with G6PD activity ≥30% to <70%

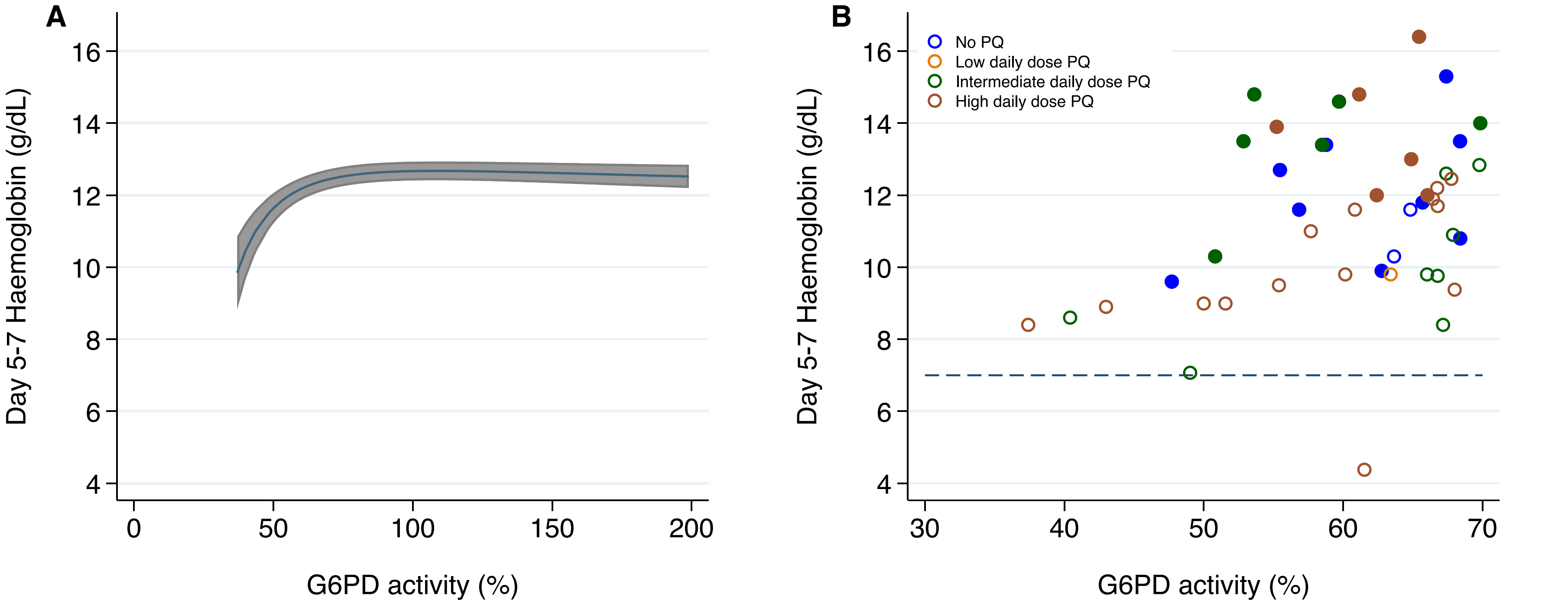

Shaded region – 95% confidence interval; Closed circles – male; open circles – female. Low daily dose is a daily primaquine dose of <0.375 mg/kg/day; Intermediate daily dose is a daily primaquine dose of 0.375 to <0.75 mg/kg/day; High daily dose is a daily primaquine dose of ≥0.75 mg/kg/day. Multivariable linear mixed-effects regression with fractional polynomial terms for G6PD activity adjusted for age, sex, day 0 parasitaemia, day 0 haemoglobin and treatment regimen with random effect for study site.

Figure S5. Observed G6PD activity versus change in haemoglobin from day 0 to A) day 2-3 and B) days 5-7

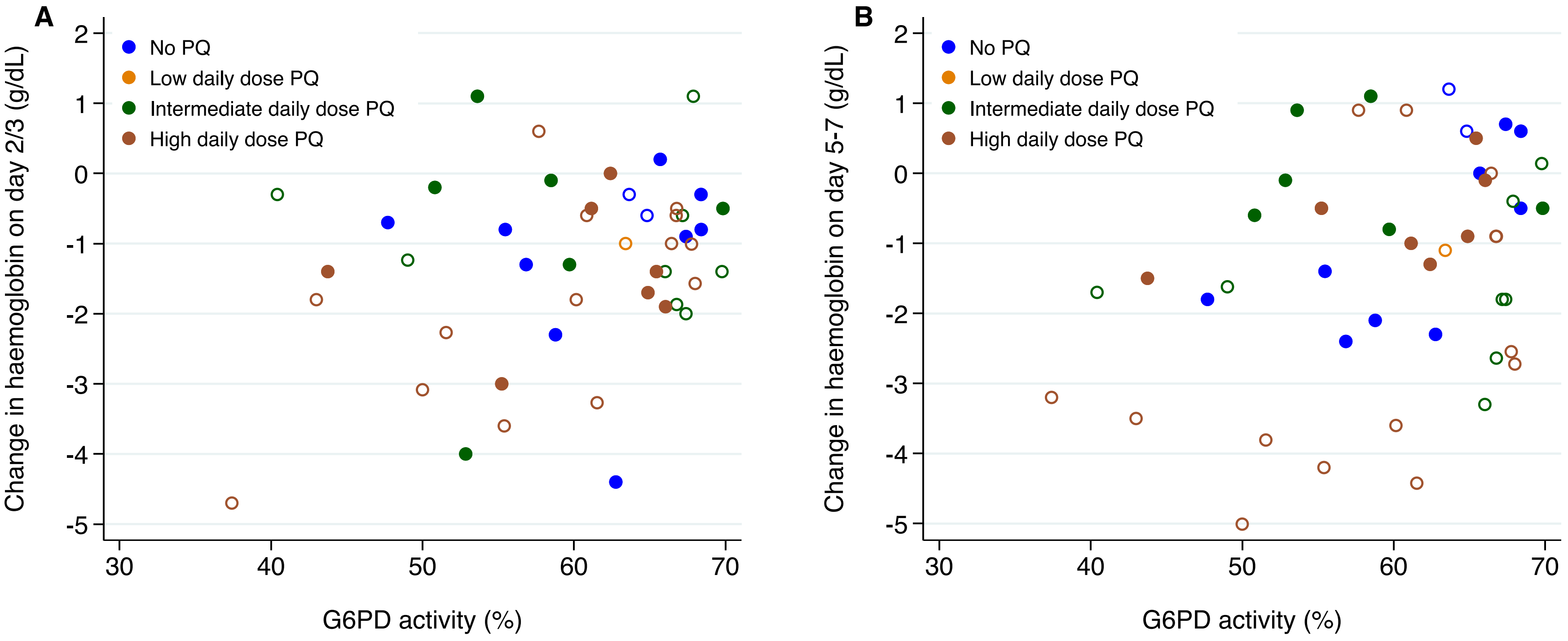

Closed circles – male; open circles – female. Low daily dose is a daily primaquine dose of <0.375 mg/kg/day; Intermediate daily dose is a daily primaquine dose of ≥0.375 to <0.75 mg/kg/day; High daily dose is a daily primaquine dose of ≥0.75 mg/kg/day.

Table S14. Haematological safety outcomes in patients with G6PD activity ≥30% to <70% and ≥70% by primaquine daily dose groups

	G6PD activity ≥30% to <70%				G6PD activity ≥70%			
	**No PQ**	**Low daily dose PQ (<0.375 mg/kg/day)**	**Intermediate daily dose PQ (≥0.375 to <0.75 mg/kg/day)**	**High daily dose PQ (≥0.75 mg/kg/day)**	**No PQ**	**Low daily dose PQ (<0.375 mg/kg/day)**	**Intermediate daily dose PQ (≥0.375 to <0.75 mg/kg/day)**	**High daily dose PQ (≥0.75 mg/kg/day)**
Hb drop of >25% to <7 g/dL								
Percentage (95% CI)	0.0 (0.0, 28.5)	0.0 (0.0, 70.8)	0.0 (0.0, 21.8)	9.1 (1.1, 29.2)	0.0 (0.0, 0.9)	0.0 (0.0, 1.3)	0.0 (0.0, 0.8)	0.2 (0.0, 1.3)
n/N	0/11	0/3	0/15	2/22	0/398	0/294	0/447	1/426
*Composite of measures of Hb change**								
Percentage (95% CI)	0.0 (0.0, 28.5)	0.0 (0.0, 70.8)	0.0 (0.0,21.8)	4.6 (0.1, 22.8)	0.0 (0.0, 0.9)	0.0 (0.0, 1.2)	0.2 (0.0, 1.2)	0.2 (0.0, 1.3)
n/N	0/11	0/3	0/15	1/22	0/398	0/312	1/467	1/426
Absolute change in Hb between day 0 and day 2-3 (g/dL), unadjusted†								
Mean (SD)	-1.1 (1.3)	-1.5 (2.6)	-0.8 (1.3)	-1.6 (1.3)	-0.5 (1.0)	-0.6 (1.0)	-0.6 (1.1)	-0.5 (1.1)
Min-Max	(-4.4, 0.2)	(-4.3, 0.8)	(-4.0, 1.1)	(-4.7, 0.6)	(-3.8, 3.1)	(-3.6, 4.0)	(-3.4, 3.9)	(-3.9, 2.9)
N	11	3	15	22	378	285	430	408
Absolute change in Hb between day 0 and days 5-7 (g/dL), unadjusted†								
Mean (SD)	-0.7 (1.4)	-0.1 (1.1)	-1.0 (1.2)	-1.8 (1.8)	-0.4 (1.0)	-0.3 (1.0)	-0.6 (1.1)	-0.5 (1.3)
Min-Max	(-2.4, 1.2)	(-1.1, 1.0)	(-3.3, 1.1)	(-5.0, 0.9)	(-4.9, 5.2)	(-3.5, 4.3)	(-5.1, 4.9)	(-5.1, 3.5)
N	11	3	15	22	377	272	424	396

*Composite measure of Hb change is a composite indicator of the presence of a haemoglobin fall below 5 g/dL or a haemoglobin fall >5 g/dL from day 0 to days 1 to 14 or renal failure needing dialysis or blood transfusion or death between days 1 to 28. † Negative values for absolute change indicate a decrease relative to baseline, while positive values indicate an increase. The 95% confidence intervals are binomial exact confidence intervals.

Table S15. Haematological safety outcomes in patients with G6PD activity ≥30% to <70% for A) female and B male patients by primaquine daily dose groups

	Female				Male			
	No PQ	Low daily dose PQ (<0.375 mg/kg/day)	Intermediate daily dose PQ (≥0.375 to <0.75 mg/kg/day)	High daily dose PQ (≥0.75 mg/kg/day)	No PQ	Low daily dose PQ (<0.375 mg/kg/day)	Intermediate daily dose PQ (≥0.375 to <0.75 mg/kg/day)	High daily dose PQ (≥0.75 mg/kg/day)
Hb drop of >25% to <7 g/dL								
Percentage (95% CI)	0.0 (0.0, 84.2)	0.0 (0.0, 84.2)	0.0 (0.0, 33.6)	13.3 (1.7, 40.5)	0.0 (0.0, 33.6)	0.0 (0.0, 97.5)	0.0 (0.0, 45.9)	0.0 (0.0, 41.0)
n/N	0/2	0/2	0/9	2/15	0/9	0/1	0/6	0/7
*Composite of measures of Hb change**								
Percentage (95% CI)	0.0 (0.0, 84.2)	0.0 (0.0, 84.2)	0.0 (0.0, 33.6)	6.7 (0.2, 32.0)	0.0 (0.0, 33.6)	0.0 (0.0, 97.5)	0.0 (0.0, 45.9)	0.0 (0.0, 41.0)
n/N	0/2	0/2	0/9	1/15	0/9	0/1	0/6	0/7
Absolute change in Hb between day 0 and day 2-3 (g/dL), unadjusted†								
Mean (SD)	-0.5 (0.2)	-0.1 (1.3)	-0.9 (1.0)	-1.7 (1.4)	-1.3 (1.4)	-4.3 (.)	-0.8 (1.7)	-1.4 (1.0)
Min-Max	(-0.6, -0.3)	(-1.0, 0.8)	(-2.0, 1.1)	(-4.7, 0.6)	(-4.4, 0.2)	(-4.3, -4.3)	(-4.0, 1.1)	(-3.0, -0.0)
N	2	2	9	15	9	1	6	7
Absolute change in Hb between day 0 and days 5-7 (g/dL), unadjusted†								
Mean (SD)	0.9 (0.4)	-0.6 (0.6)	-1.6 (1.0)	-2.4 (1.9)	-1.0 (1.2)	1.0 (.)	0.0 (0.8)	-0.7 (0.7)
Min-Max	(0.6, 1.2)	(-1.1, -0.2)	(-3.3, 0.1)	(-5.0, 0.9)	(-2.4, 0.7)	(1.0, 1.0)	(-0.8, 1.1)	(-1.5, 0.5)
N	2	2	9	15	9	1	6	7

*Composite measures of Hb change is a composite indicator of the presence of a haemoglobin fall below 5 g/dL or a haemoglobin fall >5 g/dL from day 0 to days 1 to 14 or renal failure needing dialysis or blood transfusion or death between days 1 to 28. † Negative values for absolute change indicate a decrease relative to baseline, while positive values indicate an increase. The 95% confidence intervals are binomial exact confidence intervals.

Figure S6. Sensitivity analysis restricting to G6PD activity measured during acute malaria episodes of A) the model of G6PD activity compared with haemoglobin on day 2-3 and B) observed G6PD activity versus haemoglobin on day 2-3

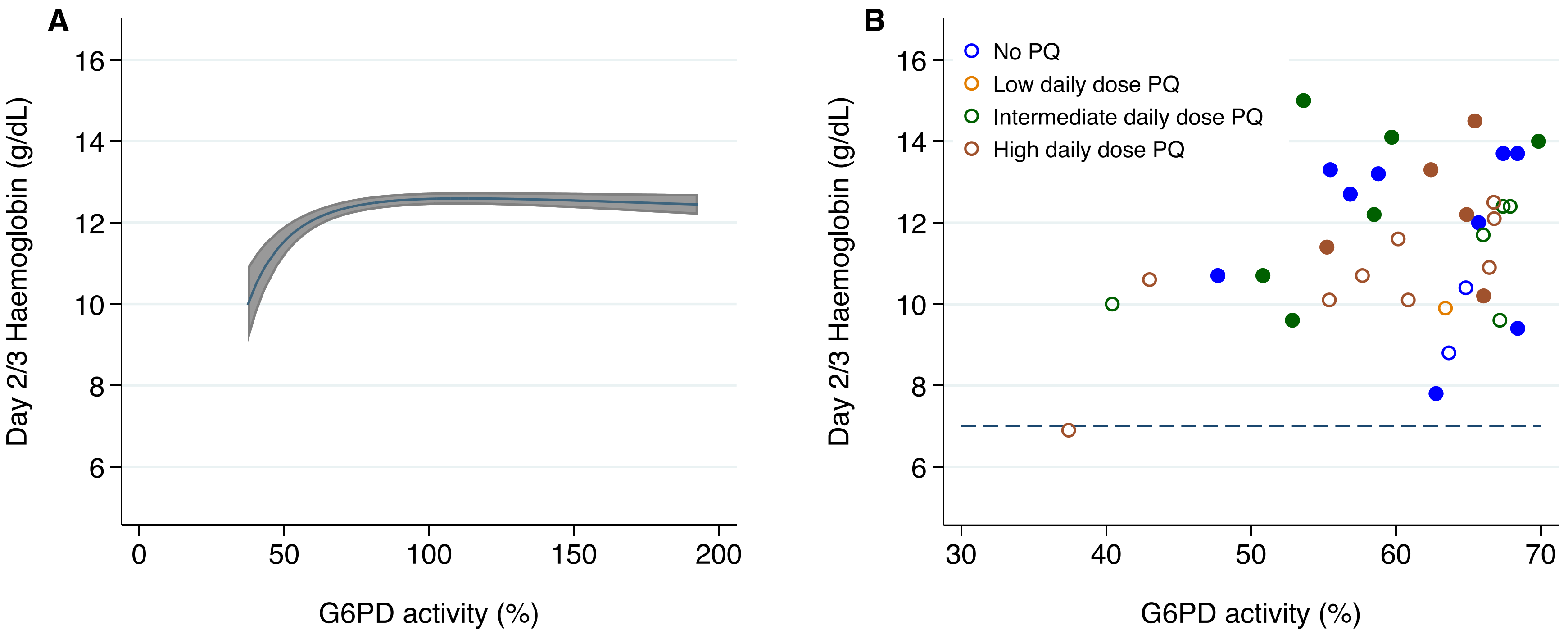

Shaded region – 95% confidence interval; Closed circles – male; open circles – female. Low daily dose is a daily primaquine dose of <0.375 mg/kg/day; Intermediate daily dose is a daily primaquine dose of ≥0.375 to <0.75 mg/kg/day; High daily dose is a daily primaquine dose of ≥0.75 mg/kg/day. Multivariable linear mixed-effects regression with fractional polynomial terms for G6PD activity adjusted for age, sex, day 0 parasitaemia, day 0 haemoglobin and treatment regimen with random effect for study site. Patients excluded if G6PD activity recorded during convalescence.

Figure S7. Sensitivity analysis restricting to G6PD activity measured during acute malaria episodes of A) the model of G6PD activity compared with haemoglobin on days 5-7 and B) observed G6PD activity versus haemoglobin on days 5-7

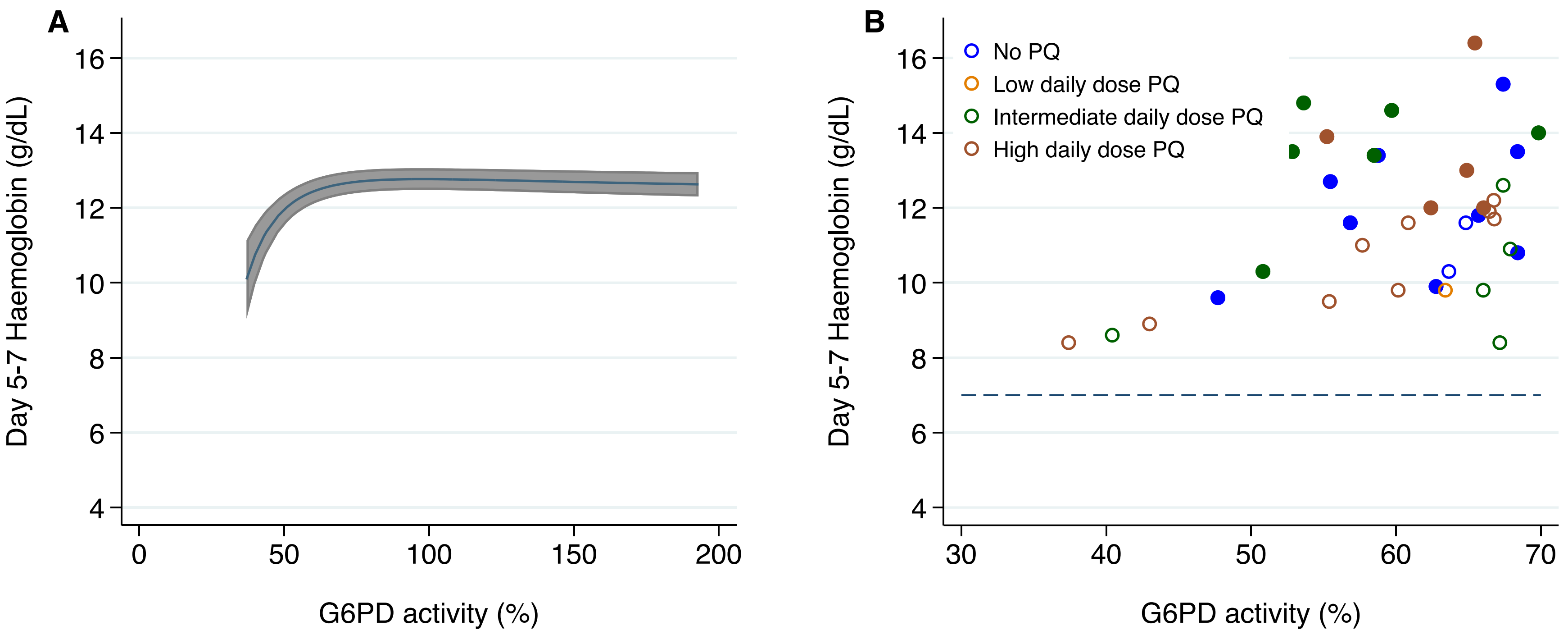

Shaded region – 95% confidence interval; Closed circles – male; open circles – female. Low daily dose is a daily primaquine dose of <0.375 mg/kg/day; Intermediate daily dose is a daily primaquine dose of ≥0.375 to <0.75 mg/kg/day; High daily dose is a daily primaquine dose of ≥0.75 mg/kg/day. Multivariable linear regression with fractional polynomial terms for G6PD activity adjusted for age, sex, day 0 parasitaemia, day 0 haemoglobin and treatment regimen with random effect for study site. Patients excluded if G6PD activity recorded during convalescence.

Figure S8. Model of G6PD activity compared with haemoglobin on day 2-3 in patients not treated with primaquine with quantitative G6PD activity ≥30%

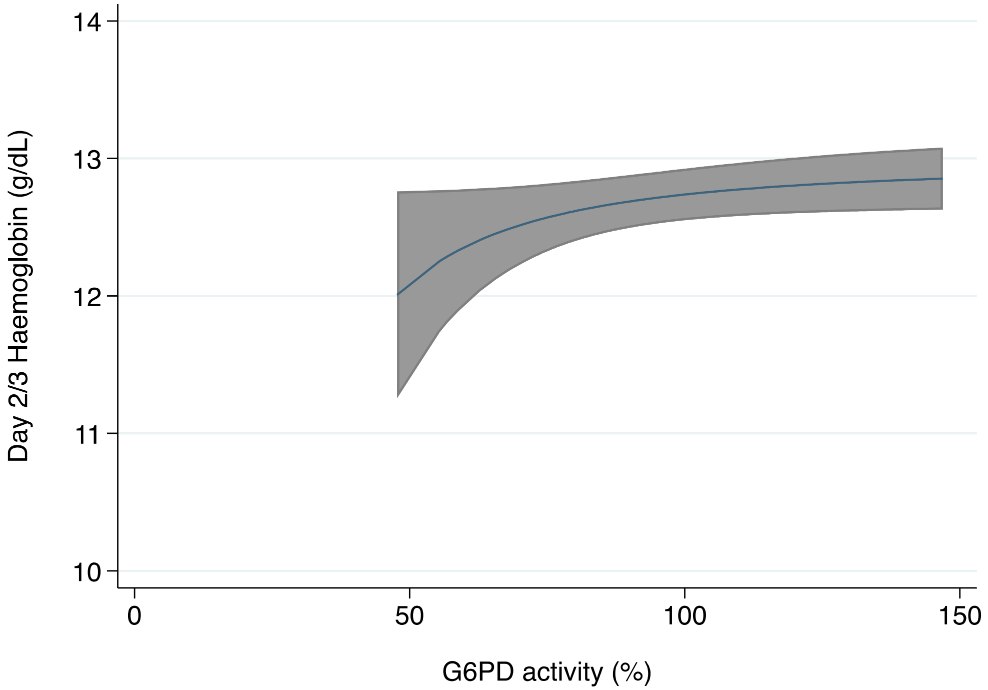

Shaded region – 95% confidence interval; Multivariable linear mixed-effects regression with fractional polynomial terms for G6PD activity adjusted for age, sex, day 0 parasitaemia and day 0 haemoglobin with random effect for study site.

References S1. Studies not included in analysis

49. Pukrittayakamee S, Chantra A, Simpson JA, et al. Therapeutic responses to different antimalarial drugs in vivax malaria. *Antimicrob Agents Chemother* 2000; **44**(6): 1680-5.

50. Carmona-Fonseca J. Vivax malaria in children: Recurrences with standard total dose of primaquine administered in 3 vs. 7 days *Iatreia* 2010; **23**(1): 10-20.

51. Saravu K, Acharya V, Kumar K, Kumar R. Plasmodium vivax remains responsive to chloroquine with primaquine treatment regimen: a prospective cohort study from tertiary care teaching hospital in southern India. *Trop Doct* 2012; **42**(3): 163-4.

52. Dharmawardena P, Rodrigo C, Mendis K, et al. Response of imported malaria patients to antimalarial medicines in Sri Lanka following malaria elimination. *PLoS One* 2017; **12**(11): e0188613.

53. Poespoprodjo JR, Burdam FH, Candrawati F, et al. Supervised versus unsupervised primaquine radical cure for the treatment of falciparum and vivax malaria in Papua, Indonesia: a cluster-randomised, controlled, open-label superiority trial. *Lancet Infect Dis* 2022; **22**(3): 367-76.

54. Mekonnen DA, Abadura GS, Behaksra SW, et al. Treatment of uncomplicated Plasmodium vivax with chloroquine plus radical cure with primaquine without G6PDd testing is safe in Arba Minch, Ethiopia: assessment of clinical and parasitological response. *Malar J* 2023; **22**(1): 135.

55. Moore BR, Salman S, Tobe R, et al. Short-course, high-dose primaquine regimens for treatment of liver-stage vivax malaria in children. *Int J Infect Dis* 2023.

56. Sutanto I, Soebandrio A, Ekawati LL, et al. Tafenoquine co-administered with dihydroartemisinin-piperaquine for the radical cure of Plasmodium vivax malaria (INSPECTOR): a randomised, placebo-controlled, efficacy and safety study. *Lancet Infect Dis* 2023.

57. Mohapatra MK, Padhiary KN, Mishra DP, Sethy G. Atypical manifestations of Plasmodium vivax malaria. *Indian J Malariol* 2002; **39**(1-2): 18-25.

58. Walsh DS, Wilairatana P, Tang DB, et al. Randomized trial of 3-dose regimens of tafenoquine (WR238605) versus low-dose primaquine for preventing Plasmodium vivax malaria relapse. *Clin Infect Dis* 2004; **39**(8): 1095-103.

59. Daneshvar C, Davis TM, Cox-Singh J, et al. Clinical and parasitological response to oral chloroquine and primaquine in uncomplicated human Plasmodium knowlesi infections. *Malar J* 2010; **9**: 238.

60. Negreiros S, Farias S, Viana GM, et al. Efficacy of Chloroquine and Primaquine for the Treatment of Uncomplicated Plasmodium vivax Malaria in Cruzeiro do Sul, Brazil. *Am J Trop Med Hyg* 2016; **95**(5): 1061-8.

61. Rajgor DD, Gogtay NJ, Kadam VS, et al. Efficacy of a 14-day primaquine regimen in preventing relapses in patients with Plasmodium vivax malaria in Mumbai, India. *Trans R Soc Trop Med Hyg* 2003; **97**(4): 438-40.

62. Ganguly S, Saha P, Guha SK, et al. In vivo therapeutic efficacy of chloroquine alone or in combination with primaquine against vivax malaria in Kolkata, West Bengal, India, and polymorphism in pvmdr1 and pvcrt-o genes. *Antimicrob Agents Chemother* 2013; **57**(3): 1246-51.

63. Macareo L, Lwin KM, Cheah PY, Yuentrakul P, Miller RS, Nosten F. Triangular test design to evaluate tinidazole in the prevention of Plasmodium vivax relapse. *Malar J* 2013; **12**: 173.

64. Tasanor O, Ruengweerayut R, Sirichaisinthop J, Congpuong K, Wernsdorfer WH, Na-Bangchang K. Clinical-parasitological response and in-vitro sensitivity of Plasmodium vivax to chloroquine and quinine on the western border of Thailand. *Trans R Soc Trop Med Hyg* 2006; **100**(5): 410-8.

65. Leslie T, Mayan I, Mohammed N, et al. A randomised trial of an eight-week, once weekly primaquine regimen to prevent relapse of plasmodium vivax in Northwest Frontier Province, Pakistan. *PLoS One* 2008; **3**(8): e2861.

66. Saravu K, Tellapragada C, Kulavalli S, et al. A pilot randomized controlled trial to compare the effectiveness of two 14-day primaquine regimens for the radical cure of vivax malaria in South India. *Malar J* 2018; **17**(1): 321.
